# Maternal Smoking During Pregnancy Induces Persistent Epigenetic Changes Into Adolescence, Independent of Postnatal Smoke Exposure and Is Associated With Cardiometabolic Risk

**DOI:** 10.3389/fgene.2019.00770

**Published:** 2019-09-05

**Authors:** Sebastian Rauschert, Phillip E. Melton, Graham Burdge, Jeffrey M. Craig, Keith M. Godfrey, Joanna D. Holbrook, Karen Lillycrop, Trevor A. Mori, Lawrence J. Beilin, Wendy H. Oddy, Craig Pennell, Rae-Chi Huang

**Affiliations:** ^1^Telethon Kids Institute, University of Western Australia, Perth, WA, Australia; ^2^Centre for Genetic Origins of Health and Disease, The University of Western Australia and Curtin University, Perth, WA, Australia; ^3^School of Pharmacy and Biomedical Sciences, Curtin University, Bentley, WA, Australia; ^4^Human Development and Health, Faculty of Medicine, University of Southampton, Southampton, United Kingdom; ^5^Early Life Epigenetics Group, MCRI, Royal Children’s Hospital, Flemington Road, Parkville, VIC, Australia; ^6^Centre for Molecular and Medical Research, School of Medicine, Deakin University, Geelong, VIC, Australia; ^7^MRC Lifecourse Epidemiology Unit and NIHR Southampton Biomedical Research Centre, University of Southampton and University Hospital Southampton NHS Foundation Trust, Southampton, United Kingdom; ^8^Centre for Biological Sciences, Faculty of Natural and Environmental Sciences, University of Southampton, Southampton, United Kingdom; ^9^Medical School, Royal Perth Hospital Unit, University of Western Australia, Perth, WA, Australia; ^10^Menzies Institute for Medical Research, University of Tasmania, Hobart, TAS, Australia; ^11^University of Newcastle, Newcastle, NSW, Australia

**Keywords:** DNA methylation, maternal smoking during pregnancy, epigenetics, Raine Study, cardiometabolic health, adolescence

## Abstract

**Background:** Several studies have shown effects of current and maternal smoking during pregnancy on DNA methylation of CpG sites in newborns and later in life. Here, we hypothesized that there are long-term and persistent epigenetic effects following maternal smoking during pregnancy on adolescent offspring DNA methylation, independent of paternal and postnatal smoke exposure. Furthermore, we explored the association between DNA methylation and cardiometabolic risk factors at 17 years of age.

**Materials and Methods:** DNA methylation was measured using the Illumina HumanMethylation450K BeadChip in whole blood from 995 participants attending the 17-year follow-up of the Raine Study. Linear mixed effects models were used to identify differential methylated CpGs, adjusting for parental smoking during pregnancy, and paternal, passive, and adolescent smoke exposure. Additional models examined the association between DNA methylation and paternal, adolescent, and passive smoking over the life course. Offspring CpGs identified were analyzed against cardiometabolic risk factors (blood pressure, triacylglycerols (TG), high-density lipoproteins cholesterol (HDL-C), and body mass index).

**Results:** We identified 23 CpGs (genome-wide *p* level: 1.06 × 10^−7^) that were associated with maternal smoking during pregnancy, including associated genes *AHRR* (cancer development), *FTO* (obesity), *CNTNAP2* (developmental processes), *CYP1A1* (detoxification), *MYO1G* (cell signalling), and *FRMD4A* (nicotine dependence). A sensitivity analysis showed a dose-dependent relationship between maternal smoking and offspring methylation. These results changed little following adjustment for paternal, passive, or offspring smoking, and there were no CpGs identified that associated with these variables. Two of the 23 identified CpGs [cg00253568 (*FTO*) and cg00213123 (*CYP1A1*)] were associated with either TG (male and female), diastolic blood pressure (female only), or HDL-C (male only), after Bonferroni correction.

**Discussion:** This study demonstrates a critical timing of cigarette smoke exposure over the life course for establishing persistent changes in DNA methylation into adolescence in a dose-dependent manner. There were significant associations between offspring CpG methylation and adolescent cardiovascular risk factors, namely, TG, HDL-C, and diastolic blood pressure. Future studies on current smoking habits and DNA methylation should consider the importance of maternal smoking during pregnancy and explore how the persistent DNA methylation effects of *in utero* smoke exposure increase cardiometabolic risk.

## Introduction

Maternal smoking during pregnancy is associated with an increased risk in the offspring of chronic diseases including asthma, certain cancers, and cardiovascular disease in adulthood (Wakschlag et al., 2002; Hofhuis et al., 2003; DiFranza et al., 2004; Oken et al., 2005; Agrawal et al., 2010; Bhattacharya et al., 2014). Together with the association of antenatal exposure to maternal smoking with DNA methylation, this highlights the importance of the early environment on the development of diseases (Gillman, 2005; Hanson and Gluckman, 2008; Schulz, 2010; Nielsen et al., 2016). Furthermore, associations with maternal smoking during pregnancy have been associated with differential methylation of cytosine–phosphate–guanine (CpG) base pairs in newborns (Joubert et al., 2016), children (Rzehak et al., 2016), young adults (Lee et al., 2015), and middle aged adults (Sun et al., 2013).

An epigenome-wide DNA methylation meta-analysis by Joubert et al. with combined sample size of 6,685 newborns and 3,187 older children previously identified 2,965 methylated CpGs in the offspring that associated with maternal smoking during pregnancy (Joubert et al., 2016). Methylation levels at CpGs most strongly associated with maternal smoking were contained within genes also implicated in other studies including *MYO1G (myosin 1G)*, *CYP1A1 (cytochrome P450 family 1 subfamily A member 1)*, *GFI1 (growth factor independent 1 transcriptional repressor)*, *CNTNAP2 (contactin-associated protein-like 2)* (Rotroff et al., 2016; Rzehak et al., 2016; Tehranifar et al., 2018), and xenobiotics (*AHRR, aryl-hydrocarbon receptor repressor*) (Finkelstein and Jeong, 2017). These genes have been associated with tumorigenesis and metastasis (in the case of *MYO1G*, *CTNAP2*, *GFI1*, and *AHRR*), activation of compounds with carcinogenic properties (in the case of *CYP1A1* and *AHRR*), and autism (*CNTNAP2*), as well as mediating the effect of maternal smoking and birthweight (in the case of *GFI1*) (Finkelstein, 2017), thereby suggesting a possible epigenetic mechanism linking exposure to smoking during pregnancy with adverse outcomes such as obesity or cancer risk in the offspring.

Several studies have focused on the effect of current smoking on CpG methylation in adults and conclude that it strongly affects DNA methylation within the genes of *AHRR*, *GFI1*, and *MYO1G* and mediates risk of disease (Su et al., 2016; Bojesen et al., 2017; Wilson et al., 2017; Li et al., 2018). However, the studies did not account for the influence of maternal smoking during pregnancy (Wilson et al., 2017; Li et al., 2018). The overlap of CpGs sites that associate with both current (adolescent or adult) smoking and maternal smoking during pregnancy indicates the need for caution in attributing causation to postnatal smoke exposure (Lee et al., 2015; Joubert et al., 2016; Rzehak et al., 2016). Richmond et al. (2015) for example showed associations between maternal smoking during pregnancy and CpG methylation measured at three different timepoints. *In utero* smoke exposure was associated with CpGs within *AHRR*, *MYO1G*, *CYP1A1*, and *CNTNAP2* independent of current smoking of the adolescents. Furthermore, a recent study in 40-year-old women showed associations between CpG methylation levels in *FTO (fat mass and obesity-associated protein)*, *CYP1A1*, *MYO1G*, *AHRR*, *ANPEP* (*alanyl aminopeptidase, membrane*), *ZNF536 (zinc finger protein 536)*, and *GFI1* and a history of exposure to maternal smoking *in utero*, which was independent of their own smoking status (Tehranifar et al., 2018). Socioeconomic status is potentially an important confounder in the association between CpG methylation and offspring smoking. Low socioeconomic status has, for example, been associated with both smoking in general (Rosemary et al., 2012) and offspring smoking (Gilman et al., 2003) and DNA methylation levels (McDade et al., 2019).

A recent study analyzed the association between eight CpGs, located in the *GFI1* gene region and cardiovascular health (Parmar et al., 2018). The authors found three out of the eight CpGs to be associated with maternal prenatal smoking and the remaining five to be associated with adolescents own smoking. They found the strongest associations between some of the CpGs with BMI, waist circumference, blood pressure, and triacylglycerol (TG), with the most consistent associations between CpGs and TG. This highlights the potential for maternal smoking to induce long lasting changes in association with both DNA methylation and cardiometabolic health.

The present study aimed to investigate whether there is an association between maternal smoking during pregnancy and DNA methylation in the offspring at 17 years of age and if methylation levels at the differentially methylated CpGs were associated with cardiometabolic risk factors, using data from the second generation (Gen2) of the Raine Study (www.rainestudy.org.au). We further determined if the relationships between methylation levels at these particular CpGs and maternal smoking were independent of paternal smoking, passive smoke exposure during childhood, and adolescent self-reported smoking. We hypothesized that there are long-term and persistent postnatal epigenetic effects following maternal smoking during pregnancy on adolescent offspring DNA methylation, relatively unaffected by smoke exposure from other sources. Furthermore, we hypothesize that these changes are associated with an adverse effect on cardiometabolic health.

## Methods

### Study

The study design and initial characteristics of the Raine Study have been previously described (Newnham et al., 1993). From 1989 to 1999, a total of 2,900 pregnant women were enrolled to take part in this longitudinal cohort study. Recruitment took part at King Edward Memorial Hospital and surrounding private hospitals. The 2,868 live births (Gen2) have been followed up at 1, 2, 3, 5, 8, 10, 14, and 17 years during which anthropometric (e.g., height, weight, skinfolds), clinical, and biochemical data have been collected.

Ethics approval for conducting the epigenetic analysis at the 17-year follow-up was given by the Human Ethics Committee of the University of Western Australia. Informed and written consent was provided by the participants and their parents or carer.

The present analyses included 790 participants that had data for the variables of interest, being maternal educational level, family income, gestational weight gain, gestational age, maternal age, maternal prepregnancy BMI, birthweight, age, Caucasian ethnicity, and sex of the child. In separate analyses, we examined the potential confounding effects of offspring smoking (*n* = 663), passive smoking (*n* = 513), and paternal smoking (*n* = 781).

### Smoking Variables

Maternal self-reported smoking during the 18th and 34th week of gestation and paternal smoking behavior (reported by the mother) at the 18th week of gestation were obtained by questionnaire. Smoking behavior of the adolescents at 17 years of age was self-reported in a confidential online questionnaire. Adolescents self-reported cigarette consumption over their lifetime (yes/no), in the past month (yes/no), and past 7 days (yes/no). Different smoking variables were derived for the analyses: Maternal smoking during pregnancy was coded as “never” versus “ever” smoking during pregnancy, based on the categorical variables for the number of cigarettes smoked daily at 18 and 34 weeks of gestation. Furthermore, maternal smoking at 18 and 34 weeks of gestation was analyzed in association with CpG in two separate statistical models, to ascertain if smoking during mid- or late-term pregnancy had a different effect on CpG methylation in the offspring. Smoking of the offspring was coded as a binary variable (“never” vs. “ever” smoked). Passive smoking exposure during childhood was defined by aggregating questionnaire data from the caregiver on smoking at eight intervening time points until 17 years (1, 2, 3, 5, 8, 10, 14, and 17) and coded as “never” versus “ever” exposed to passive smoking if the average number of cigarettes smoked over all time points was ≥1 (Le-Ha et al., 2013).

### Cardiovascular and Anthropometric Variables

Height was measured using a stadiometer (Holtain, Crosswell, UK) to the nearest 0.1 cm. Weight was measured using a digital chair scale (Wedderburn, New South Wales, Australia) to the nearest 100 g. Body mass index (BMI) was calculated as weight (kilograms)/height (meters)^2^. Waist circumference was measured to the nearest centimeter.

Venous blood samples were taken after an overnight fast. Serum insulin, glucose, high-density lipoprotein cholesterol (HDL-C), low-density lipoprotein cholesterol (LDL-C), and triacylglycerols (TG) were measured in the PathWest Laboratory at Royal Perth Hospital as described (Huang et al., 2012). HOMA-IR (molar units) was calculated by insulin (mIU/L) × glucose (mmol/l)/22.5 (Matthews et al., 1985).

### Laboratory Measures

#### DNA Methylation Profiling

Using whole-blood samples collected at age 17 years, epigenome-wide DNA methylation profiles for 1,192 (58 technical replicates) individuals were generated at the Centre for Molecular Medicine and Therapeutics, University of British Columbia using the Illumina Infinium HumanMethylation450 BeadChip array (Illumina San Diego, CA). Quality control was performed using the statistical software R and Bioconductor packages *shinyMethyl* (Fortin et al., 2014), *MethylAid* (Van Iterson et al., 2014), and *RnBeads* (Assenov et al., 2014).

Four participants with inconsistent results and identified as outliers (*n* = 3) or sex misclassification (*n* = 1) were removed. Sixty-five CpGs for which a common SNP disrupted the site leading to genotypic specific methylation levels, 11,648 sex chromosome CpGs and 10,777 CpGs with a detection *p* > 0.05 in any sample were removed. A further 160 probes with bead counts <3 in more than 5% of samples were removed. Batch effects persisted after beta-mixture quantile normalization (BMIQ) was applied (Teschendorff et al., 2013). Therefore, plate, slide, and well number were included in all statistical models. As cellular heterogeneity can influence methylation profiles and drive some of the methylation differences detectable across individual blood samples, we adjusted for estimated cell counts using the Houseman estimating method (Houseman et al., 2014) as implemented in the R statistical package, *minfi* (Aryee et al., 2014) for six cell types (CD8T, CD4T, NK, B cell, monocytes, and granulocytes). Mapping of the CpG to the nearest gene was performed using the Illumina Infinium annotation genomic coordinates.

#### Genome-Wide Genotype Data

DNA was collected from blood samples from 74% of the adolescents who attended the 14-year follow-up and a further 5% who attended the 16-year follow-up, using standardized procedures. SNP data for this study were obtained from genome-wide genotype data as described previously (Jones et al., 2013). Briefly, genotyping was performed on the Illumina Human 660W Quad Array (Illumina, San Diego, California, USA), and exclusion criteria were low genotyping success (>3% missing), excessive heterozygosity, relation with another sample (identity by descent > 0.1875), ambiguous sex, and mislabeling. There were 1,494 individuals whose DNA samples passed the quality control criteria and were eligible for genetic analyses, and 965 of them had completed the AQ. Out of those, 753 samples overlapped with the 790 samples that had epigenetic information and nonmissing data in the covariates.

### Statistical Analysis

**Figure 1:**
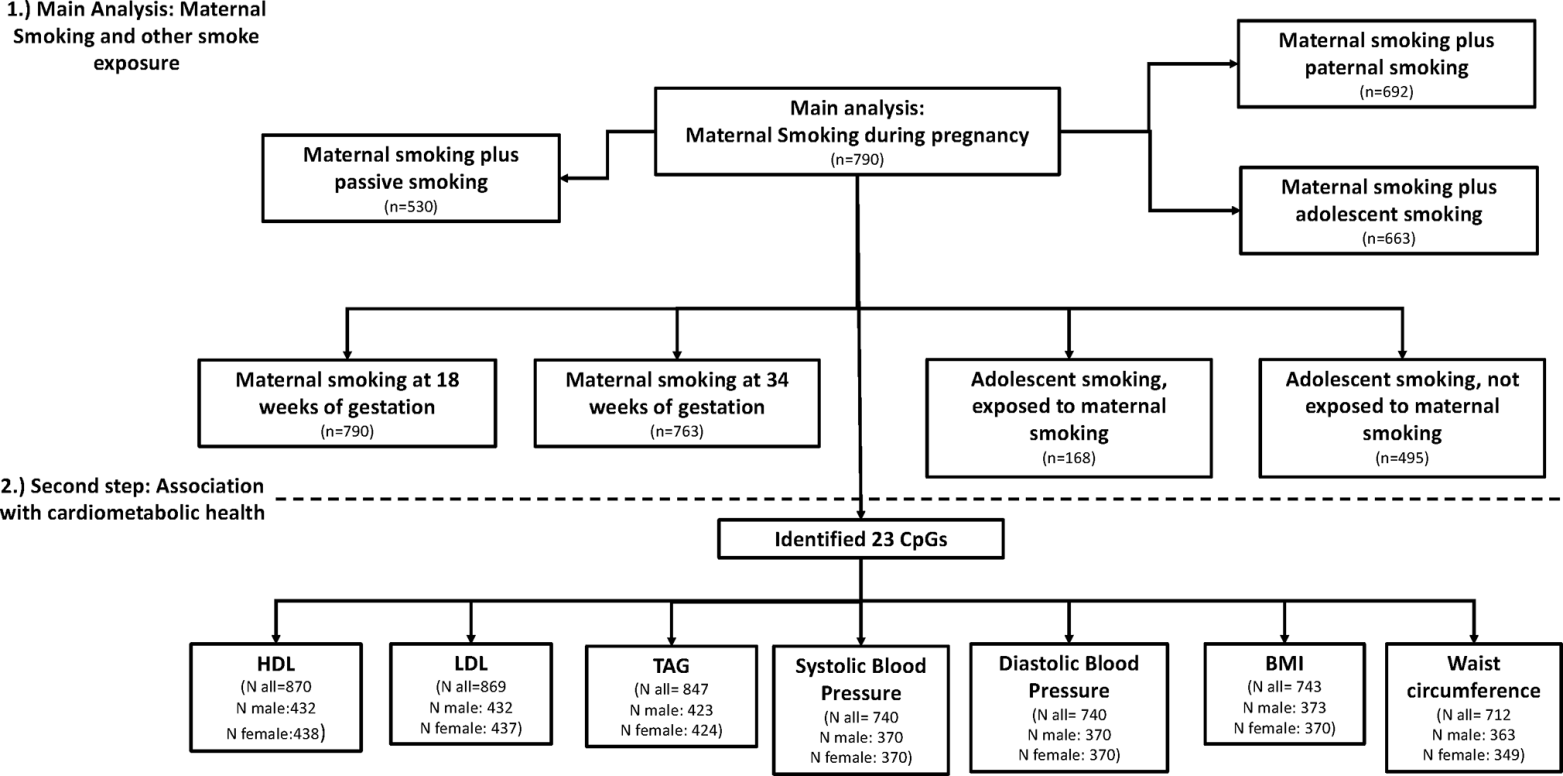
Flow diagram of the analysis and sample size per model.

All models were analyzed using R Version 3.4.3. A flow diagram of models and sample size is presented in **Figure 1**, showing the different analysis steps and the number of complete cases per analysis. Full results are presented in the **Supplemental Tables**.

#### Effect Modifier

Studies previously conducted to analyze the association between maternal smoking during pregnancy and offspring CpG methylation levels have controlled for a variety of effect modifiers with a high level of commonalities between studies. Potential maternal confounders included prepregnancy BMI and socioeconomic variables including family income and maternal education, gestational weight gain, maternal alcohol consumption during pregnancy, and maternal age. Offspring variables included birthweight, sex, and age. Analyses were adjusted for cell count using the reference-based Houseman approach (Sun et al., 2013), for batch effect and measurement derived variability utilizing linear mixed effect models with plate number as the random effect and the aforementioned variables. Hypothesis testing for differences between the maternal smoking exposed and the nonexposed group in the variables used in this study were performed using chi-squared tests for categorical and *t*-tests and Wilcoxon tests for continuous variables.

#### Models

##### Identification of CpGs Whose Methylation Levels Associated With Maternal Smoking

We used a linear mixed-effects model as our main model. The outcome was percent methylation at one single CpG per model, and the predictor was maternal smoking, adjusted for exact age of the adolescent, sex, and maternal, and offspring confounders as described previously (*n* = 790). The same model was run twice, with the second application also adjusted for blood cell count estimate (Houseman et al., 2014). This stepwise approach ensures that differences in the adjustment for cell count are accessible. To account for multiple testing, we utilized a conservative Bonferroni approach (genome-wide *p* level: 1.06 × 10^−7^).

The main model with maternal smoking was then assessed for a sex interaction to examine if the effect of maternal smoking on the CpG methylation levels differed between male and female.

Additionally, we used the information on maternal average cigarette consumption during pregnancy (six categories: none, 1–5 daily; 6–10 daily; 11–15 daily; 16–20 daily; and 31 or more, with none as the reference group) to test for dose-dependent methylation in the identified sites.

##### Assessing the Effect of Paternal, Passive, and Adolescent Smoking on DNA Methylation

We included the covariates “ever smoking at 17 years of age” (yes/no) (*n* = 663), paternal smoking during pregnancy (*n* = 781) and passive smoking exposure during childhood (*n* = 513) (Le-Ha et al., 2013) to the main model predicting the CpGs associated with maternal smoking, to ascertain if they changed the effect size of maternal smoking. We also examined the effect of those variables in separate models as predictors.

For adolescent smokers, we split the data into those who were exposed to maternal smoking during pregnancy (*n* = 168) and those who were not exposed (*n* = 495) to explore any potential differences in effect sizes. The model was the same as described above, including confounders as described earlier.

##### Assessing the Genetic Effect on the Association Between Maternal Smoking and CpG Methylation

To assess if the association between maternal smoking and CpG methylation persists after taking SNPs into account, we utilized the *GEM* bioconductor package (Pan et al., 2016) to identify significant SNP–CpG associations in the Raine study. The *GEM* package is a computational efficient approach to identify methylation quantitative trait loci, perform DNA-methylation wide association studies, and assess the interaction of CpG methylation and SNPs on outcomes.

With *GEM*, those SNPs were identified, which were significantly associated with the CpGs associated with maternal smoking during pregnancy in this study. The identified SNPs were added to the respective main model with CpG as outcome and maternal smoking during pregnancy as predictor, adjusted for the aforementioned variables.

## Results

### Characteristics of the Population

The characteristics of the participants are shown in **Table 1**. Of the 995 Caucasian participants, 30% were exposed to maternal smoking during pregnancy. Paternal, passive, and adolescent smoking rates were higher in the group exposed to maternal smoking. Of those exposed to maternal smoking during pregnancy, 60% had fathers who also smoked during the pregnancy period, 41% were exposed to passive smoking, and 30% reported smoking themselves. Of those not exposed to maternal smoking, 24% had fathers who smoked during the pregnancy period, 25% were exposed to passive smoking, and 40% reported smoking themselves.

**Table 1:** Characteristics of the participants of the Raine Study. The *p* value refers to chi-square test results for categorical and *t*-test/Wilcoxon test for continuous variables.

	No maternal smoking	Maternal smoking	*p* value
N	699	296	
**ADOLESCENT CHARACTERISTICS**			
**Sex of the child [** ***n*** **(%)]**			0.17
Female	337 (48.21)	157 (53.04)	
Male	362 (51.79)	139 (46.96)	
**Adolescent age**			0.133
Mean	17.24	17.29	
SD	0.58	0.61	
**Birthweight**			2.09E−11
Mean	3,430.85	3,137.17	
SD	526.64	657.46	
**Corrected gestational age (days)**			0.00071
Mean	276.59	272.52	
SD	12.18	19.31	
**Waist circumference (cm)**			0.039
Mean	79.03	81.15	
SD	11.11	12.55	
**Adolescent BMI (kg/m²)**			0.001
Mean	22.8	24.12	
SD	4.16	5.19	
**Adolescent HDL-C (mg/dl)**			0.03337
Mean	1.3	1.27	
SD	0.29	0.3	
**Adolescent LDL-C (mg/dl)**			0.35
Mean	2.33	2.38	
SD	0.66	0.71	
**Adolescent triacylglycerol (mg/dl)**			0.012
Mean	1.04	1.15	
SD	0.5	0.67	
**Adolescent systolic blood pressure (mmHg)**			0.3247
Mean	114.99	115.69	
SD	11.37	10.73	
**Adolescent diastolic blood pressure (mmHg)**			0.6639
Mean	59.43	59.6	
SD	6.56	6.78	
**Adolescent HOMA-IR**			0.1503
Mean	2.04	2.11	
SD	2.85	1.82	
**SMOKE EXPOSURE DURING** **OTHER PERIODS OF LIFE**			
**Paternal smoking during pregnancy [** ***n*** **(%)]**			<2.2E−16
No	519 (74.25)	113 (38.18)	
Yes	174 (24.89)	178 (60.135)	
Missing	6 (0.86)	5 (1.69)	
**Passive smoking [n(%)]**			<2.2E−16
No	292 (41.77)	20 (6.76)	
Yes	134 (19.17)	122 (41.22)	
Missing	273 (39.06)	154 (52.03)	
**Adolescent smoking**			0.002541
Yes	381 (54.51)	119 (40.20)	
No	158 (22.60)	75 (25.34)	
Missing	160 (22.89)	102 (34.46)	
**MATERNAL AND FAMILIAL** **CHARACTERISTICS**			
**Maternal alcohol consumption during pregnancy [** ***n*** **(%)]**			0.4107
no	354 (50.64)	147 (49.66)	
yes	344 (49.21)	149 (50.34)	
missing	1 (0.14)		
**Maternal school level [** ***n*** **(%)]**			1.65E−11
None	292 (41.77)	182 (61.49)	
Trade certificate or apprenticeship	55 (7.87)	27 (9.12)	
Professional registration (nondegree)	90 (12.88)	22 (7.43)	
College diploma or degree	129 (18.45)	40 (13.51)	
University degree	102 (14.59)	10 (3.38)	
Other	31 (4.43)	15 (5.07)	
**Family income [** ***n*** **(%)]**			2.91E-06
<24,000 AUD per annum	206 (29.47)	127 (42.91)	
>24,000 AUD per annum	475 (67.95)	148 (50)	
NA	18 (2.58)	21 (7.09)	
**Maternal age (years)**			1.62E−12
Mean	29.17	26.76	
SD	5.8	5.63	
**Maternal prepregnancy BMI (kg/m²)**			0.3591
Mean	22.66	22.25	
SD	4.45	4.51	
**Maternal pregnancy weight gain ratio**			0.9944
Mean	0.5	0.5	
SD	0.2	0.21	

Offspring exposed to maternal smoking during pregnancy included 49% from families in the lowest income bracket compared with 29% of those not exposed to maternal smoking. The group exposed to maternal smoking during pregnancy had significantly more mothers with a lower educational attainment (*p* < 0.001). Mothers who smoked during pregnancy were younger (26.76 ± 5.63 vs. 29.17 ± 5.8 years old) than those who did not smoke.

Those exposed to maternal smoking had significantly higher waist circumference (*p* = 0.039), BMI, and TG and lower HDL-C at 17 years of age compared to the nonexposed group.

For all smoking variables, those study participants of the original cohort who are not included in this study (*n* = 1,317) had higher numbers of smokers and smoke exposed individuals as well as lower socioeconomic status assessed by family income compared to the participants included in our study [*n* = 995 (**Supplement Table S1**)].

### Epigenome-Wide DNA Methylation Analysis

#### Effects of Maternal Smoking on Offspring DNA Methylation

One identified CpG, namely, cg04224247 (*WWC3*), showed a bimodal distribution in the histogram, suggestive of a genotype driven rather than an environmental influence (Teh et al., 2014) and hence was excluded from further analysis.

**Figure 2:**
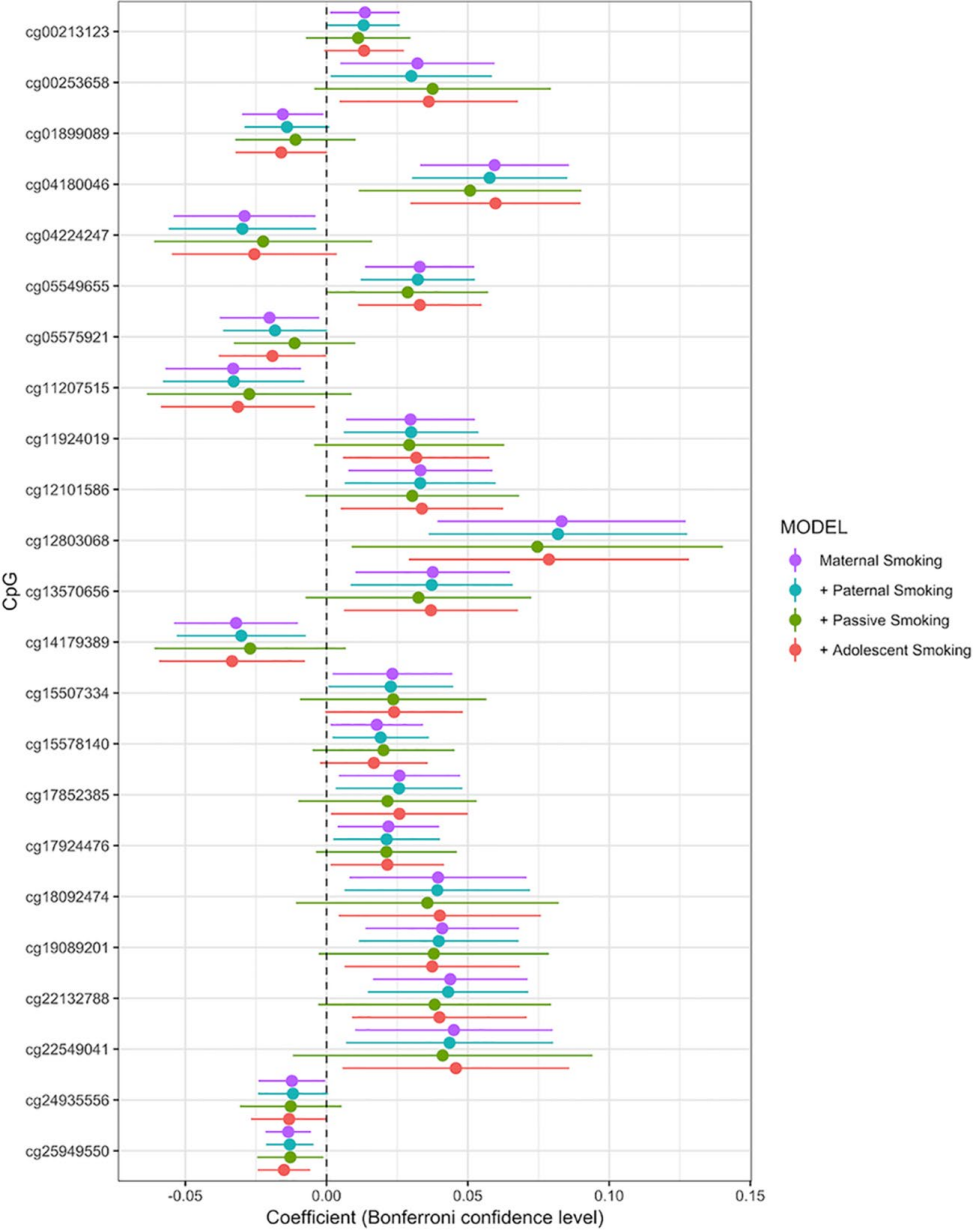
Forrest plot for the epigenome-wide association study with maternal smoking as predictor and individual CpG sites as outcome, adjusted for sex, offspring age, age of the mother, birthweight, gestational weight gain, maternal alcohol consumption during pregnancy, maternal school level, maternal prepregnancy BMI, family income during pregnancy, cell count, and batch effects. Stratified by models without further adjustment (*n* = 790), adjustment for paternal smoking (*n* = 692), passive smoking (*n* = 530), and adolescent smoking (*n* = 663). *X-axis*: effect size from the linear mixed effects model and confidence interval; *Y-axis*: individual CpGs.

Associations between any maternal smoking during pregnancy and methylation levels at individual CpG sites are shown in a forest plot (**Figure 2**). The analysis showed that inclusion or exclusion of cell count estimation based on the Houseman method did not change the number of CpGs whose methylation levels were associated with maternal smoking after Bonferroni correction (genome-wide *p* level: 1.06 × 10^−7^, **Supplemental Tables S2**, **S3**, and **S4**). The smoking variables that combined data from 18 and 34 weeks did not differ in the direction of their association with CpGs in that methylation at the same CpGs was associated with maternal smoking at i) 18 weeks, ii) 34 weeks, and iii) combined 18 or 34 weeks (**Supplemental Tables S1**, **S5**, and **S6**).

The final model, including all confounding variables and batch number as random effect, showed methylation levels at 23 CpGs associated with maternal smoking during pregnancy after conservative Bonferroni correction; these 23 CpGs mapped to 10 genes (**Table 2**). Overall, seven CpGs (genes: *CNTNAP2*, *GFI1*, *WWC3*, *AHRR*, and *APOB*) showed hypomethylation in association with mothers who reported they were smoking during pregnancy, whereas 16 CpGs (associated genes: *CYP1A1/CYP1A2*, *MIR548T*, *AHRR*, *FRMD4A*, *FTO*, and *MYO1G*) were hypermethylated in those whose mothers smoked during pregnancy compared to offspring of nonsmokers. The highest percentage difference in DNA methylation between maternal smoking categories was 8.3% at cg12803068 (mapped to *MYO1G*). The remainder of the top 23 CpGs show methylation changes in association with *in utero* smoke exposure in the range of 1–6% (**Table 2**, coefficient times 100).

**Table 2:** CpGs associated with maternal smoking during pregnancy with UCSC gene annotation and model p-values, beta-coefficients and standard errors from the epigenome wide association study. The Bonferroni significance threshold is 1.06 × 10^–7^.

Chromosome	ID	Function	Gene	Location	UCSC_CpG_Islands_Name	Relation_to_UCSC_CpG_Island	P.Value	Coefficient	Standard Error
7	cg04180046	intronic	MYO1G	Body	chr7:45002111-45002845	Island	8.93E-31	0.059451122	0.004899729
7	cg12803068	intronic	MYO1G	Body	chr7:45002111-45002845	S_Shore	1.04E-22	0.083162901	0.008174077
15	cg05549655	intergenic	CYP1A1,CYP1A2	TSS1500	chr15:75018186-75019336	Island	5.83E-19	0.032945838	0.003593891
7	cg25949550	intronic	CNTNAP2	Body	chr7:145813030-145814084	S_Shore	1.39E-18	–0.013562513	0.001496779
7	cg22132788	exonic	MYO1G	Body	chr7:45002111-45002845	Island	5.43E-17	0.043777558	0.005088082
7	cg19089201	UTR3	MYO1G	3’UTR	chr7:45002111-45002845	Island	2.76E-15	0.040922837	0.005057333
1	cg14179389	intronic	GFI1	Body	chr1:92945907-92952609	Island	1.53E-14	–0.032050625	0.004077831
7	cg11207515	intronic	CNTNAP2	Body			3.80E-13	–0.033065175	0.004462166
15	cg13570656	intergenic	CYP1A1,CYP1A2	TSS1500	chr15:75018186-75019336	Island	4.33E-13	0.037564996	0.005082323
15	cg11924019	intergenic	CYP1A1,CYP1A2	TSS1500	chr15:75018186-75019336	Island	6.07E-12	0.029699312	0.004240665
15	cg12101586	intergenic	CYP1A1,CYP1A2	TSS1500	chr15:75018186-75019336	Island	6.16E-12	0.033228312	0.004745949
15	cg22549041	intergenic	CYP1A1,CYP1A2	TSS1500	chr15:75018186-75019336	Island	1.02E-11	0.045041658	0.00650389
15	cg18092474	intergenic	CYP1A1,CYP1A2	TSS1500	chr15:75018186-75019336	Island	2.95E-11	0.03943739	0.005831697
5	cg17924476	intronic	AHRR	Body	chr5:320788-323010	S_Shore	1.16E-10	0.021888448	0.003342621
15	cg17852385	intergenic	CYP1A1,CYP1A2	TSS1500	chr15:75018186-75019336	Island	2.05E-10	0.025807923	0.003996831
16	cg00253658	intergenic	FTO, LOC100996338				4.50E-10	0.032150112	0.005079598
X	cg04224247	intronic	WWC3	5’UTR	chrX:9982513-9984583	Island	9.01E-10	–0.029045508	0.004673566
5	cg05575921	intronic	AHRR	Body	chr5:373842-374426	N_Shore	1.26E-09	–0.020194441	0.003278542
15	cg00213123	intergenic	CYP1A1,CYP1A2	TSS1500	chr15:75018186-75019336	Island	5.06E-09	0.013568664	0.002291017
10	cg15507334	upstream	FRMD4A	TSS200			5.57E-09	0.023297316	0.003944714
7	cg15578140	ncRNA_intronic	MIR548T	Body			8.41E-09	0.017756893	0.003043603
5	cg01899089	intronic	AHRR	Body	chr5:370185-370422	N_Shore	1.06E-08	–0.015519815	0.002679093
2	cg24935556	intergenic	APOB, LOC645949				3.37E-08	–0.012281317	0.002198374

Inclusion or exclusion of cell count estimation in the model did not change either the number or direction of association CpGs falling under the Bonferroni threshold (genome-wide *p* level: 1.06 × 10^−7^, **Supplemental Tables S2** and **S3**).

#### Dose Dependency With Maternal Smoking: Sensitivity Analysis

A sensitivity analysis for dose-dependent methylation with maternal smoking showed a significant trend towards hyper- or hypomethylation with an increasing number in cigarettes consumed (Wilcoxon significance test between groups *p* < 0.05). These results can be seen in the supplement (**Supplemental Figure 1**).

#### Effect of Other Sources of Smoke Exposure on Offspring DNA Methylation

Adjusting the main maternal smoking model for all additional smoking variables reduced the sample size due to missing values in the offspring self-reported smoking to 506 but did not change the direction of the estimates, as can be seen in the forest plot, comparing the models by beta coefficients (**Supplemental Figure 2** and **Supplemental Table S7**). Methylation levels at four CpGs (cg04180046, cg12803068, cg25949550, and cg05549655) were still significantly associated with maternal smoking (**Supplemental Table S6** and **S7**). These were also the most significant CpGs in the main model.

Stratified analysis for methylation levels at the identified 23 CpGs for male and female did not show significant effect size changes compared to the full model (**Supplemental Figure 3**). Furthermore, **Supplemental Figure 4** shows a barplot comparing the methylation change between those not exposed to any smoking (*n* = 381), those only smoking at 17 years (*n* = 158), those only exposed to maternal smoking during pregnancy (*n* = 119), and those exposed to both types of smoking (*n* = 75).

#### Effect of Paternal Smoking During Pregnancy

Paternal smoking (yes/no) during pregnancy (*n* = 692) was not significantly associated with methylation levels at any of the 23 CpGs detected in the maternal smoking EWAS (all genome-wide *p* > 1.06 × 10^−7^, **Supplemental Table S8**). Adding paternal smoking to the main model did not change the effect size for the effect of maternal smoking on CpG methylation levels. An EWAS of paternal smoke exposure did not detect any associations with CpG methylation levels at the genome-wide significance *p* < 1.06 × 10^−7^ (**Supplemental Table S8**).

#### Effect of Childhood Exposure to Smoking

No significant associations were detected for passive smoke exposure of the adolescent (*n* = 530) (Le-Ha et al., 2013) with DNA methylation at any CpG (all genome-wide *p* > 1.06 × 10^−7^) (**Table 2**, **Supplemental Table S9**). Adding passive childhood exposure to the main model did not change the effect size for the effect of maternal smoking on CpG methylation levels. An EWAS of passive smoke exposure did not show any associations with CpG methylation levels at the genome-wide significance *p* < 1.06 × 10^−7^ (**Supplemental Table S9**).

#### Effect of Adolescent Smoking

**Figure 3:**
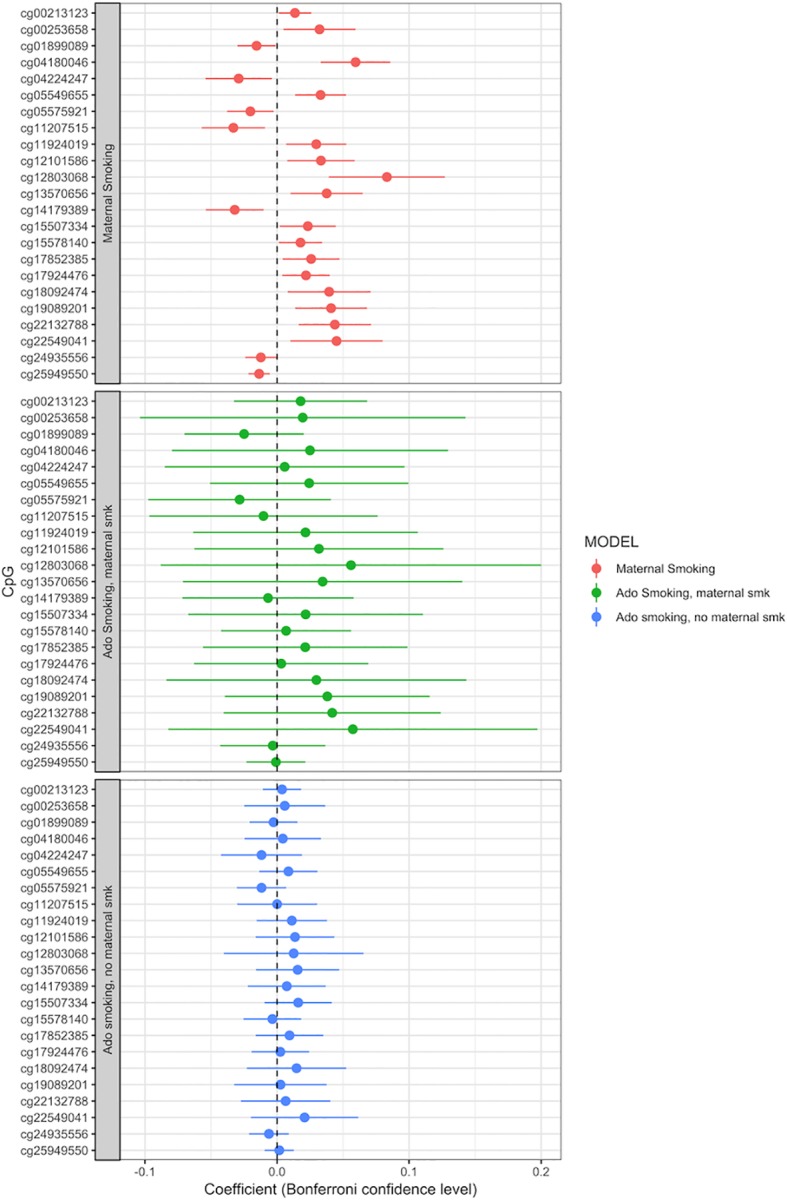
Adolescent smoking, stratified by exposure to maternal smoking during pregnancy, and compared to the effect of maternal smoking during pregnancy (maternal smoking *n* = 790, adolescent smoking, maternal smoking *n* = 168, adolescent smoking, no maternal smoking *n* = 495). Models adjusted for sex, offspring age, age of the mother, birthweight, gestational weight gain, maternal alcohol consumption during pregnancy, maternal school level, maternal prepregnancy BMI, family income during pregnancy, cell count, and batch effects. *X-axis*: effect size from the linear mixed effects model and confidence interval; *Y-axis*: individual CpGs.

Adolescent reported smoking behavior (yes/no, *n* = 663) was not associated with the methylation level of any of the 23 CpGs detected in the maternal smoking EWAS (full results of the adolescent smoking EWAS: **Supplemental Table S10**). The *p* values, effect sizes and standard errors are reported in **Table 2**. Adding active adolescent smoking to the main model did not change the effect size for the effect of maternal smoking on CpG methylation. An EWAS of adolescent smoking did not detect any associations with CpG methylation levels at genome-wide significance *p* < 1.06 × 10^−7^ (**Supplemental Table S9**). We performed a stratified analysis for adolescents who were smoking, but were not exposed to maternal smoking during pregnancy, versus those who were exposed. Comparison of the beta coefficients suggested a stronger effect of maternal smoking than of adolescent smoking; for the group of not exposed adolescent smokers, the beta coefficients were smaller than the ones for those exposed to maternal smoking, but none of the CpGs were significantly associated with adolescent smoking (**Figure 3**, **Supplemental Tables S11** and **S12**).

#### Cardiometabolic Variables

Analyses that examined the methylation levels at the 23 CpGs associated with maternal smoking and cardiometabolic risk factors in the entire study population and separately for male and female, showed methylation levels at two CpGs (cg00253568 and cg00213123, located in the *FTO* and *CYP1A1* region) significantly associated with TG (cg00253568, full study population; coefficient, 1.97; standard error, 0.63; Bonferroni *p* value, 0.041), diastolic blood pressure (cg00253568, female subset; coefficient, 3.06; standard error, 0.91; Bonferroni *p* value, 0.021), and HDL-C (cg00213123, male subset; coefficient, 6.72; standard error, 2.04; Bonferroni *p* value, 0.025), whereas almost all of the 23 CpGs showed trends of either hyper- or hypomethylation in association with cardiometabolic variables, indicating a potentially long lasting effect of maternal smoking on cardiometabolic health of the offspring (Boxplots for cardiometabolic variables, stratified by exposure to maternal smoking: **Supplemental Table S5**).

#### Effect of SNPs on the Association Between CpG Methylation and Maternal Smoking

When adding the SNPs that were associated with the identified 23 CpGs in this study to the main model, the significant association between CpG methylation and maternal smoking persisted (**Supplemental Table S13**). Furthermore, the SNPs were not significantly associated with exposure to maternal smoking during pregnancy. This means that the association between DNA methylation and maternal smoking during pregnancy seems to be independent of SNPs, highlighting the potential importance of environmental influences on DNA methylation.

## Discussion

In this study, we showed associations between *in utero* maternal smoking exposure and CpG methylation in whole-blood DNA from adolescents, independent of paternal smoking during the period of pregnancy, cumulative passive smoke exposure, and adolescent smoking. Additionally, we showed a trend for dose-dependent effects of maternal smoking on offspring CpG methylation levels. The CpG methylation level associations with maternal smoking are in accordance with previous findings at birth (Joubert et al., 2016), during childhood (Rzehak et al., 2016), adolescence (Lee et al., 2015), and in middle age (Sun et al., 2013). Apart from cg24935556 (*APOB*), all CpGs identified in this study were identified in the meta-analysis by Joubert et al. (2016).

We did not detect associations between paternal smoking during pregnancy, adolescent smoking, or passive smoking exposure and DNA methylation. Our findings suggest that maternal smoking during pregnancy induces long-lasting DNA methylation changes in the offspring established by adolescence, which are not greatly modified by postnatal smoke exposure. Furthermore, we found that methylation levels at two CpGs (cg00253568 and cg00213123, located in the *FTO* and *CYP1A1* region) identified in association with maternal smoking during pregnancy were also associated with cardiometabolic health variables, suggesting that maternal smoking during pregnancy may induce changes that affect offspring cardiometabolic health.

### Maternal Smoking

Differential hypermethylation associated with *AHRR*, *CNTNAP2*, *CYP1A1*, *FRMD4A*, *GFI1*, *MYO1G*, and *CYP1A1* has been shown previously in the same CpGs that we identified (Rotroff et al., 2016; Tehranifar et al., 2018). A study analyzing the associations between maternal smoking during pregnancy and adolescent CpG methylation levels in a discovery population of 132 and a replication cohort of *n* = 447 also showed methylation levels associated to maternal smoking within *MYO1G*, *CNTANAP2*, *GFI1*, *CYP1A1*, and *AHRR* but did not analyze the effect of any other sources of smoke exposure on CpG methylation levels (Lee et al., 2015). The majority of CpG sites in the meta-analysis by Joubert et al. (2016) were identified with the same direction of effect as in our study. Given Joubert et al. analyzed cord blood and our study used whole blood, these findings demonstrate consistent methylation patterns over different tissue types and time of sampling, which suggests lasting effects of maternal smoking during pregnancy on offspring DNA methylation. The specific methylation sites that we identified are consistent with previous reports in neonates (from cord blood) and middle-age populations [whole blood, lymphocytes (mononuclear)] (Philibert et al., 2013; Zeilinger et al., 2013). This establishes a high consistency of DNA methylation markers related to maternal smoking during pregnancy. Such lifetime persistence and consistency are essential prerequisites for using DNA methylation as a valid biomarker for exposure and potentially a predictor for future adverse health outcomes. Therefore, this study fills the gap in the literature confirming stable changes in DNA methylation in adolescence.

Overall, the findings from our study suggest that methylation changes are induced in early life and persist into adolescence. Maternal smoking during pregnancy potentially exposes the fetus to cigarette-related chemicals and toxins leading to an early life “programming” effect that persists into adolescence and potentially affects long-term health.

### Paternal Smoking Effects

There are fewer studies examining the effect of paternal than maternal smoking on offspring health, although prepregnancy exposure to paternal smoking is associated with a higher risk of leukemia, childhood cancers, and asthma in the offspring (Jenkins et al., 2017).

Analyses in the ALSPAC study suggested associations between paternal prepregnancy smoking and offspring BMI (Pembrey et al., 2005). In this study, 166 fathers were identified who started smoking before their offspring was aged 11 years. Compared to the nonsmoking fathers and fathers with later onset of smoking, the male offspring of fathers who commenced smoking before they were 11 years old had a higher BMI at 9 years of age. When tested in the Raine study with paternal smoking during pregnancy and BMI of the offspring at 16 years of age, a significant association (*p* = 0.008) was found after adjusting for age and sex.

In another study, Jenkins et al. used the Illumina 450K BeadChip to examine the effect of paternal cigarette smoking on sperm DNA methylation (Jenkins et al., 2017) in 78 male never smokers compared to 78 smokers. They showed that 141 CpG loci were differentially methylated in the sperm of smokers and suggested transgenerational inheritance. In our study, we did not find any effects of paternal prepregnancy smoking on offspring whole-blood DNA methylation, possibly due to our sample being insufficiently statistically powered. It is also possible that the effect of paternal smoking might be less prominent and too small to detect given our sample size. However, the inability to detect associated CpG methylation at genome-wide significance, while being able to detect a large number of CpGs with methylation levels associated maternal smoking, suggests a dominant effect of maternal smoke exposure.

### Adolescent Smoking

There are some studies that have consistently shown cross-sectional associations of CpG methylation with current smoking in adults and adolescents. For example, the CpG cg05575921, mapped to *AHRR* in our study, has been associated with smoking in a recent study (Li et al., 2018). Similarly, a study that analyzed the effect of smoking on several timepoints and after smoking cessation (Wilson et al., 2017) showed that cg05575921 and five other CpGs related to *AHRR* associated with smoking. Another study by Lee et al. in a Korean population with a sample size of 100 (31 current, 30 former, and 39 never smoker) showed similar results, with the strongest association again being with cg05575921 (Lee et al., 2016).

A limitation of each of these studies that have examined the effect of adolescent smoking on DNA methylation is attributing the findings to current smoking without consideration of the possible effects of *in utero* exposure in the form of maternal smoking during pregnancy. This is very likely a complication, as offspring of maternal smokers are more likely to smoke themselves (Gilman et al., 2003; Rosemary et al., 2012). Within our study, 55% of adolescent smokers had mothers who smoked.

In the current study, we addressed this issue by running separate analyses for those adolescents who smoked themselves and were exposed to maternal smoking during pregnancy versus the ones who were not exposed. This showed that the beta coefficients in the adolescent smokers who were also exposed to maternal smoking were most similar to the CpGs associated with maternal smoking in our main analysis. Although the sample size is too small to show significant effects, this suggests a dominant effect of *in utero* smoke exposure. This tendency can be seen in **Supplemental Figure 4**, when comparing the methylation change between the study participants not exposed to any smoking, those smoking at 17 years, those exposed to maternal smoking during pregnancy, and those exposed to both types of smoking. In this barplot, the exposure to maternal smoking during pregnancy causes the majority of the methylation change, mostly equal to those exposed to maternal smoking and smoking themselves.

### Passive Smoking

To our knowledge, there are no studies to date analyzing the effect of passive smoke exposure over the life course on DNA methylation, despite the evidence that passive smoking is associated with manifold diseases such as chronic obstructive pulmonary disease, wheeze, asthma, and food allergy, as well as cancer (Le-Ha et al., 2013; Saulyte et al., 2014; Vardavas et al., 2016). A study in the Avon Longitudinal Study of Parents and Children analyzed passive smoke exposure as paternal smoking during pregnancy and mothers exposure to smoking of her father and mother but did not assess the offspring’ s postnatal passive smoke exposure (Richmond et al., 2018).

In our analysis, we did not detect associations between lifetime passive smoke exposure and CpG methylation in adolescence. The accuracy and reliability of measurement of passive exposure may be limited. However, validity is enhanced in the current study by repeated longitudinal measures, which act as internal validation and prospective collection of data. The consistent answering of the question on maternal and paternal smoking over eight follow-ups, rather than from a single time point, increases the likelihood of a valid measure.

### Cardiometabolic Risk-Related Genes

We observed increased methylation, within the *FTO* gene (cg00253658; chr16:54210496), in the offspring of mothers who had smoked during pregnancy. Variants in this gene have previously been shown to associate with birthweight and the development of obesity and diabetes (Frayling et al., 2007) Their functional impact may be in modifying expression of the *IRX3* and *IRX5* genes, rather than *FTO* itself (Smemo et al., 2014). Others have found hypermethylation in the region of this gene in relation to maternal smoking, in African American and Hispanic populations although in a different CpG, namely cg03687532 (chr16:54228358) (Tehranifar et al., 2018). Furthermore, methylation levels are *FTO*, and *CYP1A1* mapped CpGs were significantly associated with TG, diastolic blood pressure and HDL-C in our study, suggesting correlation with early life environments (i.e., smoke exposure) and later cardiometabolic health.

Another study found that CpGs associated with exposure to maternal smoking during pregnancy were also associated with all cause as well as cardiovascular mortality. This study identified significant associations for all cause and cardiovascular specific mortality with the CpGs cg05575921 and cg06126421 (Zhang et al., 2016). In our study, cg05575921 was associated with maternal smoking during pregnancy but not significantly associated with any of the cardiometabolic risk factors. However, methylation levels at cg05575921 were associated with the lowest *p* value across the genome with systolic blood pressure in the male and female combined analysis (uncorrected *p* = 0.02, Bonferroni corrected = 0.47), the lowest *p* value in the female only association with systolic blood pressure (uncorrected *p* = 0.006, Bonferroni corrected = 0.14), among the top 5 lowest *p* values in the female only with diastolic blood pressure analysis (uncorrected *p* = 0.017, Bonferroni corrected = 0.39), and methylation levels at this CpG with the second lowest *p* value in the female only analysis with TG (uncorrected *p* = 0.01, Bonferroni corrected = 0.34). Considering the low sample sizes, especially of the female subset (*n* = 370), there may be a suggestive association.

The beforementioned study by Parmar et al. (2018) found a most significant association between CpG methylation levels and maternal prenatal smoking with waist circumference, TG, and blood pressure with cg14179389 (*GFI1*). As stated previously, this CpG was also among the 23 CpGs identified as having methylation levels significantly correlated with maternal smoking in our study, but methylation levels for cg14179389 were not significantly associated with cardiometabolic risk in our analysis. For the female subset in the TG analysis, however, methylation at this CpG had the lowest uncorrected *p* value (uncorrected *p* = 0.01, Bonferroni corrected = 0.27). This sex-specific tendency seems to be in line with what Parmer et al. observed, as they stated that adjusting their model for sex, age, and adult own smoking strengthened the association. Furthermore, considering they found associations with a Bonferroni corrected *p* ≤ 0.01 within a meta-analysis, accessing a sample size of 18,212 adults, our findings only showing tendencies with a maximum of *n* = 870 is not surprising.

### Strengths and Limitations

Strengths of this study are the prospective and repeated measures (at eight time points) of cigarette smoke exposure in ∼800 participants. The internal validation of cross-checking answers across time increases the reliability of the questionnaire data. Our findings accord with the same CpG sites that associate with smoking in studies that have used cotinine levels to confirm smoking status (Joubert et al., 2012; Philibert et al., 2013; Lee et al., 2016; Morales et al., 2016; Rotroff et al., 2016). DNA methylation sites identified in our study are in gene regions previously associated with maternal smoking and are in the same direction of association (Joubert et al., 2016).

While cotinine is considered the gold standard for evaluation of smoking, a number of studies have shown very high correlations between cotinine levels and questionnaire data, up to 97% (Patrick et al., 1994; Parazzini et al., 1996; Vartiainen et al., 2002; Dolcini et al., 2003). A subset of the Raine study mothers (*n* = 238) had cotinine measures available at 28 weeks of gestation, and, as previously shown, cotinine concentration significantly differed between the groups of reported number of cigarettes smoked, highlighting the validity of the Raine study maternal smoking questionnaire data (Stick et al., 1996).

A further strength of our study is the ability to adjust for a wide range of possible confounders, in particular socioeconomic status, which is associated with smoking behavior and DNA methylation (McDade et al., 2019). However, it is still possible that other unmeasured environmental factors in pregnancy or postnatally could be influencing or modifying some of these findings. Owing to the deeply phenotyped character of the Raine study, we were able to adjust all the models for multiple sources of smoke exposure, narrowing down to the specific effect of maternal smoking on DNA methylation in the offspring. The fact that we found associations between methylation levels at the identified CpGs and cardiometabolic health-related variables suggests correlations between smoke exposure and offspring health.

A further strength of our study is that we integrated genetic (SNPs) and epigenetic (CpG methylation) information and assessed if the association between CpG methylation and maternal smoking during pregnancy still persists when accounting for SNPs. To our knowledge, this was not done to this extent in any DNA methylation wide association study previous to ours.

The majority of CpG sites in the meta-analysis by Joubert et al. (2016) were identified with the same direction of effect as in our study. Given that Joubert et al. analyzed cord blood and our study used whole blood, these findings demonstrate consistent DNA methylation patterns over different sample types and time points in response to maternal smoking during pregnancy.

A potential limitation is that we only examined methylation from whole-blood DNA, which might not be the site of change in association with smoking. Few population studies have cell-sorted DNA methylation, and our findings suggest that some of these changes may be induced across multiple cell types. Furthermore, the sample sizes of some of the analysis that we conducted are below 200, making them potentially underpowered to detect small epigenetic changes.

It is known that up to 6% of the probes in the Illumina Methylation450 BeadChip kit could give false positives, due to known cross-reactivity. Furthermore, the array only covers 2% of the epigenome CpG DNA methylation sites (Kurdyukov and Bullock, 2016). To mitigate some of these limitations, we performed thorough preprocessing and QC steps to remove any problematic probes and samples. In addition, we accounted for batch effects in all our models and used a conservative Bonferroni correction for multiple testing to minimize any false positives that may have arisen due to technical issues from probes on the 450k array.

In our cohort, it is encouraging that we show similar associations between *in utero* smoke exposure and CpG methylation, both in amount and specific sites (Breton et al., 2017). However, performing independent methylation analysis such as pyrosequencing would have further strengthened the inferences. Lastly, for the dose–response relationship, the questionnaire variable for the number of cigarettes consumed needs to be analyzed with caution. With questionnaire data, there is always a chance of recall bias or underreporting, especially when it comes to behaviors such as cigarette or alcohol consumption.

## Conclusions

We have shown associations between maternal smoking during pregnancy and offspring DNA methylation at 23 CpGs in adolescents at age 17 years. These associations were predominantly driven by maternal smoking and not modified by paternal, passive, or adolescent smoking. Furthermore, we are unable to detect genome-wide significant associations with paternal smoking and passive smoke exposure at any CpG sites. Our data that suggest DNA methylation changes in offspring are likely due to the direct effect of maternal smoking during pregnancy, rather than current, passive, or paternal smoking. Future studies on smoking habits and DNA methylation should adjust for maternal smoking, in addition to socioeconomic status of the mother and/or offspring, depending on the age of the offspring. The specific methylation sites that we identified are in agreement with previous reports in neonates (from cord blood) and middle aged populations [whole blood, lymphocytes (mononuclear)] (Philibert et al., 2013; Zeilinger et al., 2013). This establishes a high consistency of DNA methylation markers related to maternal smoking during pregnancy. Such persistence and consistency are essential prerequisites for using DNA methylation as a valid biomarker for exposure and potentially a predictor for future adverse health outcomes. Furthermore, we showed that maternal-smoking-induced methylation changes are associated with cardiometabolic variables, suggesting early life “programming” of later life cardiometabolic health.

## Data Availability

The datasets used during and/or analyzed during the current study are available from the corresponding author on reasonable request.

## Ethics Statement

Ethics approval for conducting the epigenetic analysis at the 17-year follow-up was given by the Human Ethics Committee of the University of Western Australia. Informed and written consent was provided by the participants and their parents or carer.

## Author Contributions

SR wrote the manuscript performed the analysis and interpreted the results. R-CH, PM, T-M, L-B, GB, JC, K-G, J-H, and KL contributed to the conception and design of the study, revised the manuscript and helped to interpret the results. CP and WO contributed to interpretation of the results and revised the manuscript. All authors contributed to manuscript revision, read and approved the submitted version.

## Funding

The DNA methylation work was supported by NHMRC grant 1059711. R-CH and T-M are supported by NHMRC Fellowships (grant numbers 1053384 and 1042255, respectively).

This work was supported by resources provided by The Pawsey Supercomputing Centre with funding from the Australian Government and the Government of Western Australia.

SR received support from the European LifeCycle project through the fellowship call of June 2018, Grant agreement No. 733206. K-G is supported by the UK Medical Research Council (MC_UU_12011/4), the National Institute for Health Research (as an NIHR Senior Investigator (NF-SI-0515-10042) and through the NIHR Southampton Biomedical Research Centre) and the European Union’s Erasmus + Capacity-Building ENeA^SEA^ Project and Seventh Framework Programme (FP7/2007–2013), projects EarlyNutrition and ODIN under grant agreement numbers 289346 and 613977.

RCH, PM, and SR received further support through the NHMRC EU-collaborative grant with the number APP1142858—Early life stressors and lifecycle health.

## Conflict of Interest Statement

The authors declare that the research was conducted in the absence of any commercial or financial relationships that could be construed as a potential conflict of interest.
