# Supplementary material for: Maternal Smoking During Pregnancy Induces Persistent Epigenetic Changes Into Adolescence, Independent of Postnatal Smoke Exposure and Is Associated With Cardiometabolic Risk
Source: Front Genet. 2019 Sep 5;10:770. doi: 10.3389/fgene.2019.00770 (PMC6764289; doi:10.3389/fgene.2019.00770)
Supplement: Supplementary file 14 [file DataSheet_1.pdf]

Supplemental Figure S1. Boxplots for the 23 identified CpGs, stratified by amount of cigarettes smoked during pregnancy. Y-axis: % DNA methylation.

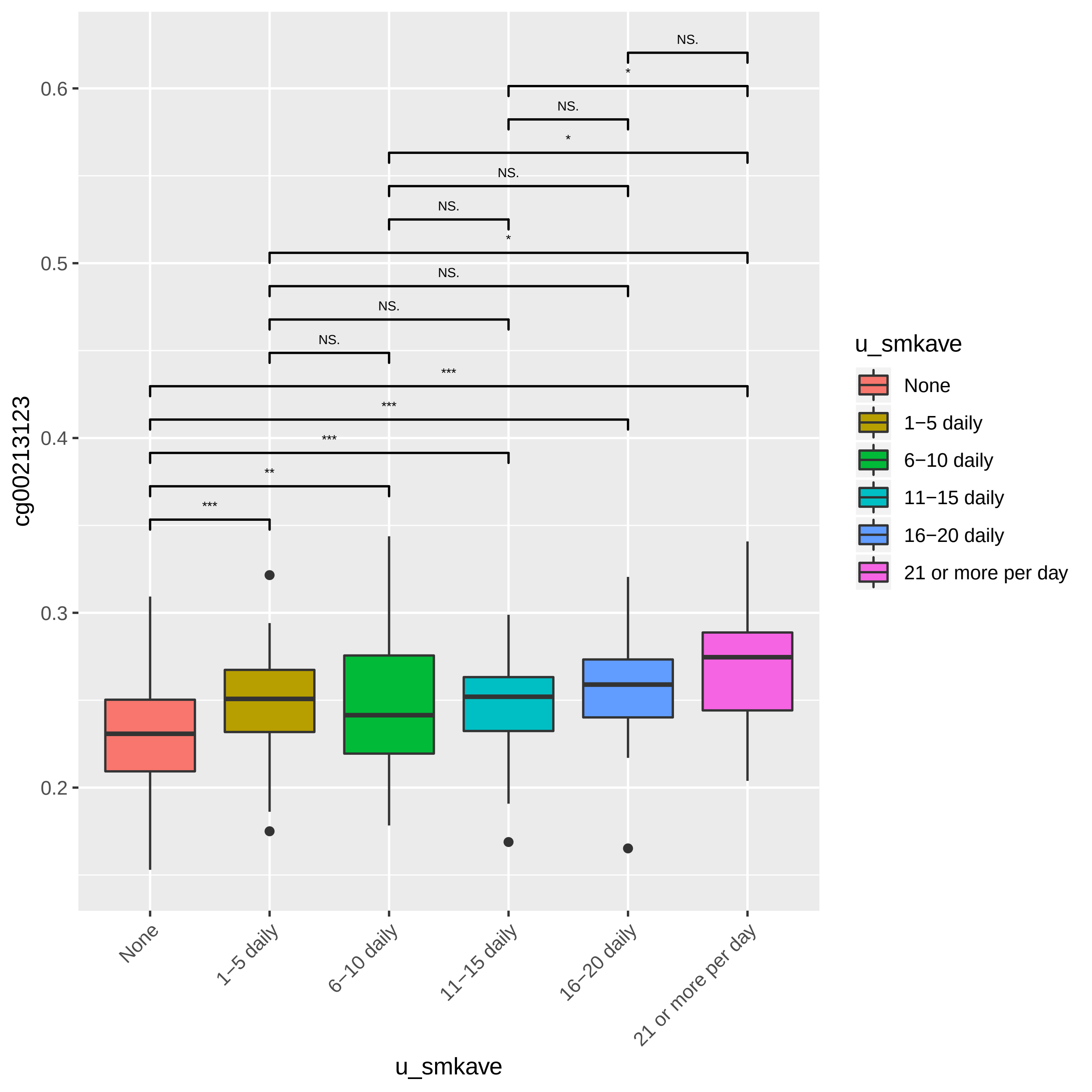
NS.
*
NS.
*
NS.
NS.
*
NS.
NS.
NS.
***
***
***
**
***
0.6
0.5
0.4
0.3
0.2
cg00213123
None
1−5 daily
6−10 daily
11−15 daily
16−20 daily
21 or more per day
u_smkave
u_smkave
None
1−5 daily
6−10 daily
11−15 daily
16−20 daily
21 or more per day

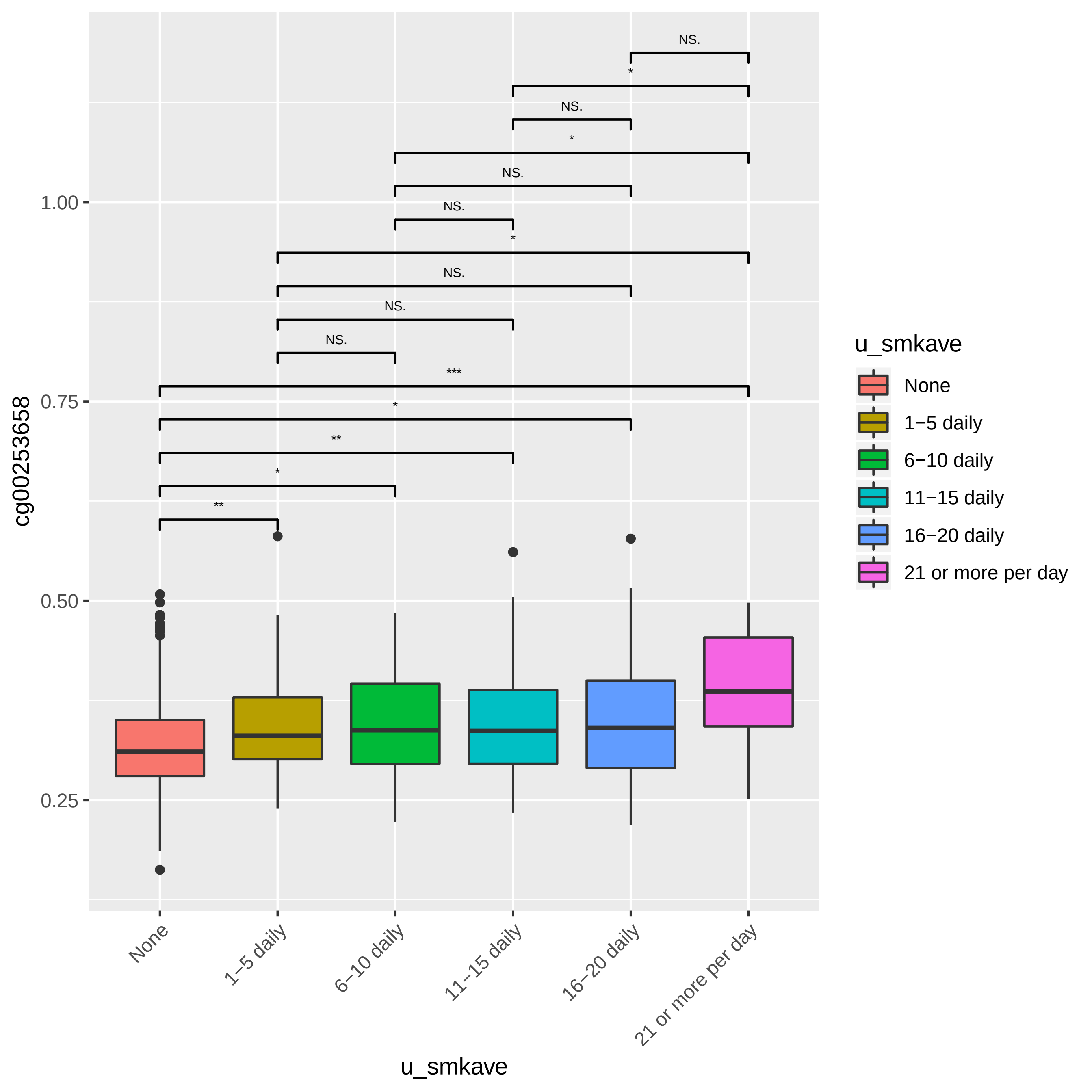
NS.
*
NS.
*
NS.
NS.
*
NS.
NS.
NS.
***
*
**
*
**
1.00
0.75
0.50
0.25
cg00253658
None
1−5 daily
6−10 daily
11−15 daily
16−20 daily
21 or more per day
u_smkave
u_smkave
None
1−5 daily
6−10 daily
11−15 daily
16−20 daily
21 or more per day

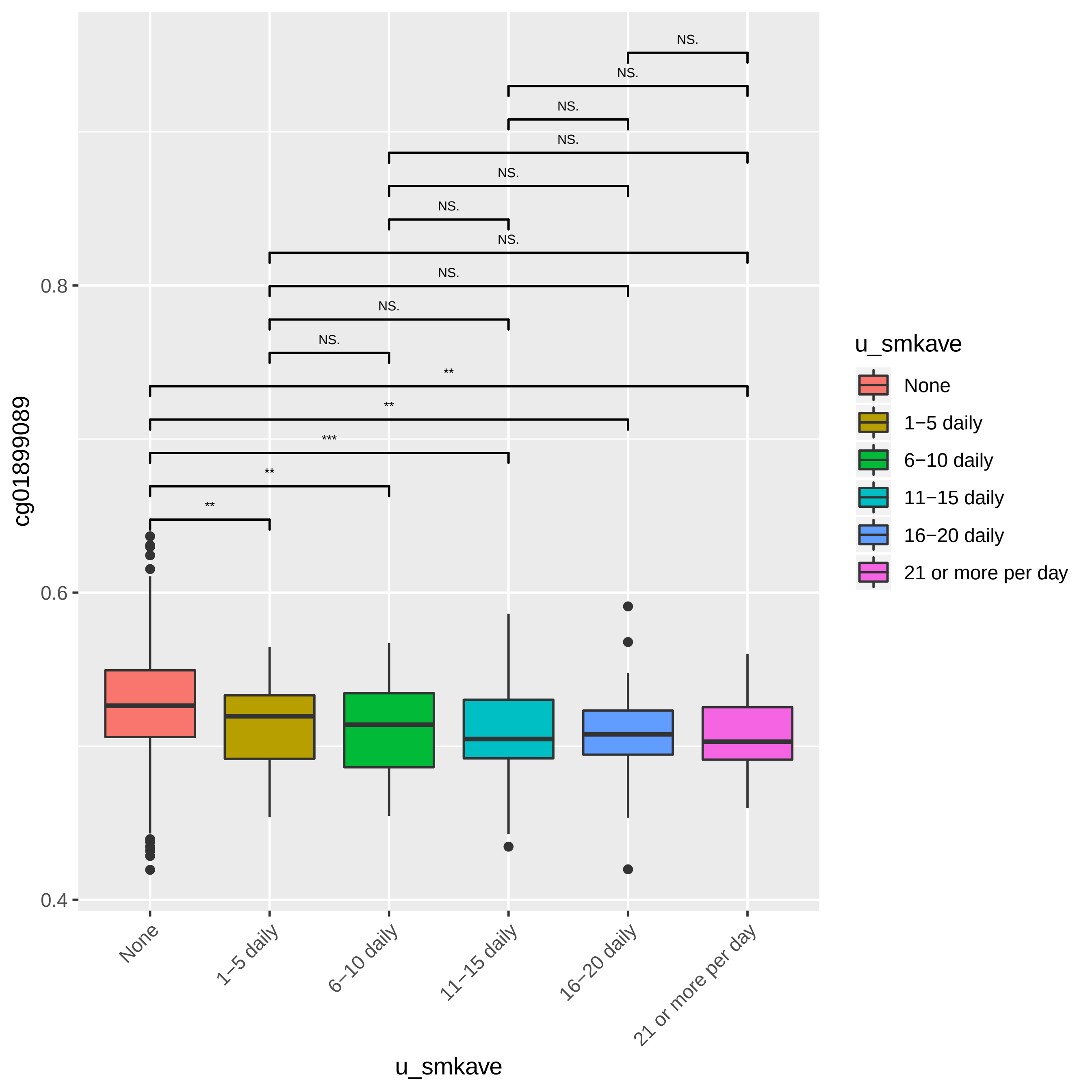
NS.
NS.
NS.
NS.
NS.
NS.
NS.
NS.
NS.
NS.
**
**
***
**
**
0.8
0.6
0.4
cg01899089
None
1−5 daily
6−10 daily
11−15 daily
16−20 daily
21 or more per day
u_smkave
u_smkave
None
1−5 daily
6−10 daily
11−15 daily
16−20 daily
21 or more per day

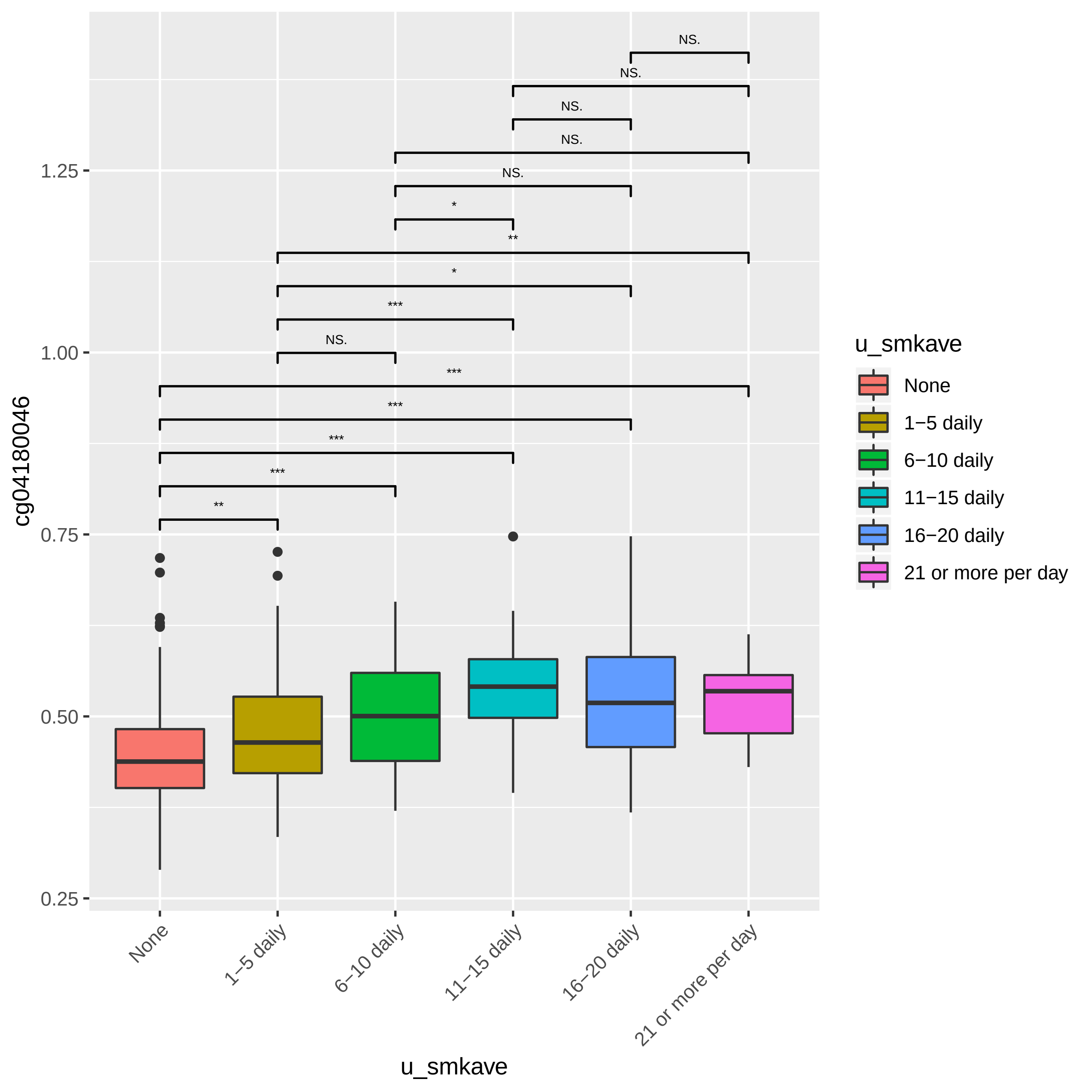

NS.
NS.
NS.
NS.
NS.
*
**
*
***
NS.
***
***
***
***
**
1.25
1.00
0.75
0.50
0.25
cg04180046
None
1−5 daily
6−10 daily
11−15 daily
16−20 daily
21 or more per day
u_smkave
u_smkave
None
1−5 daily
6−10 daily
11−15 daily
16−20 daily
21 or more per day

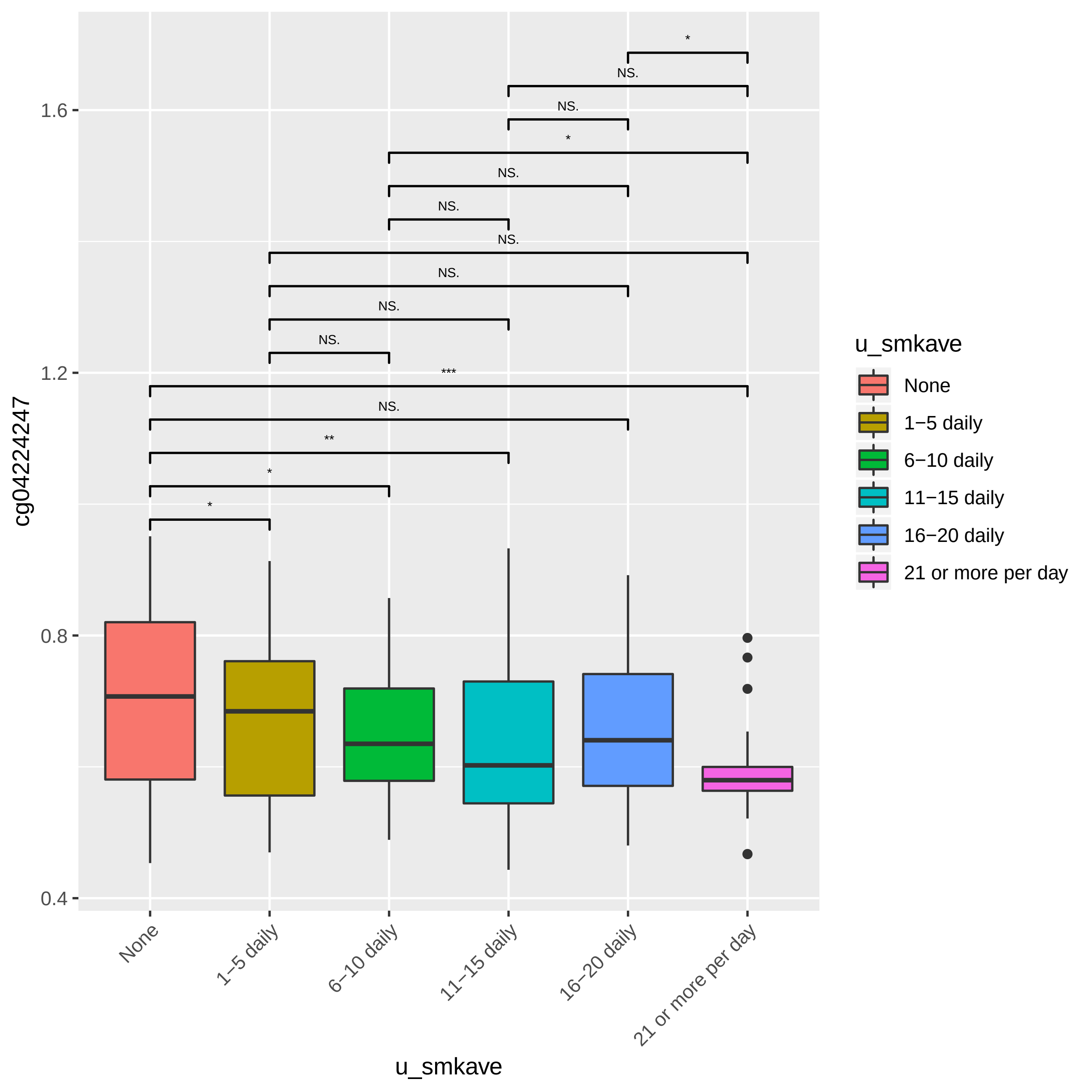

u_smkave
None
1−5 daily
6−10 daily
11−15 daily
16−20 daily
21 or more per day
cg04224247
1.6
1.2
0.8
0.4
NS.
*
**
***
u_smkave

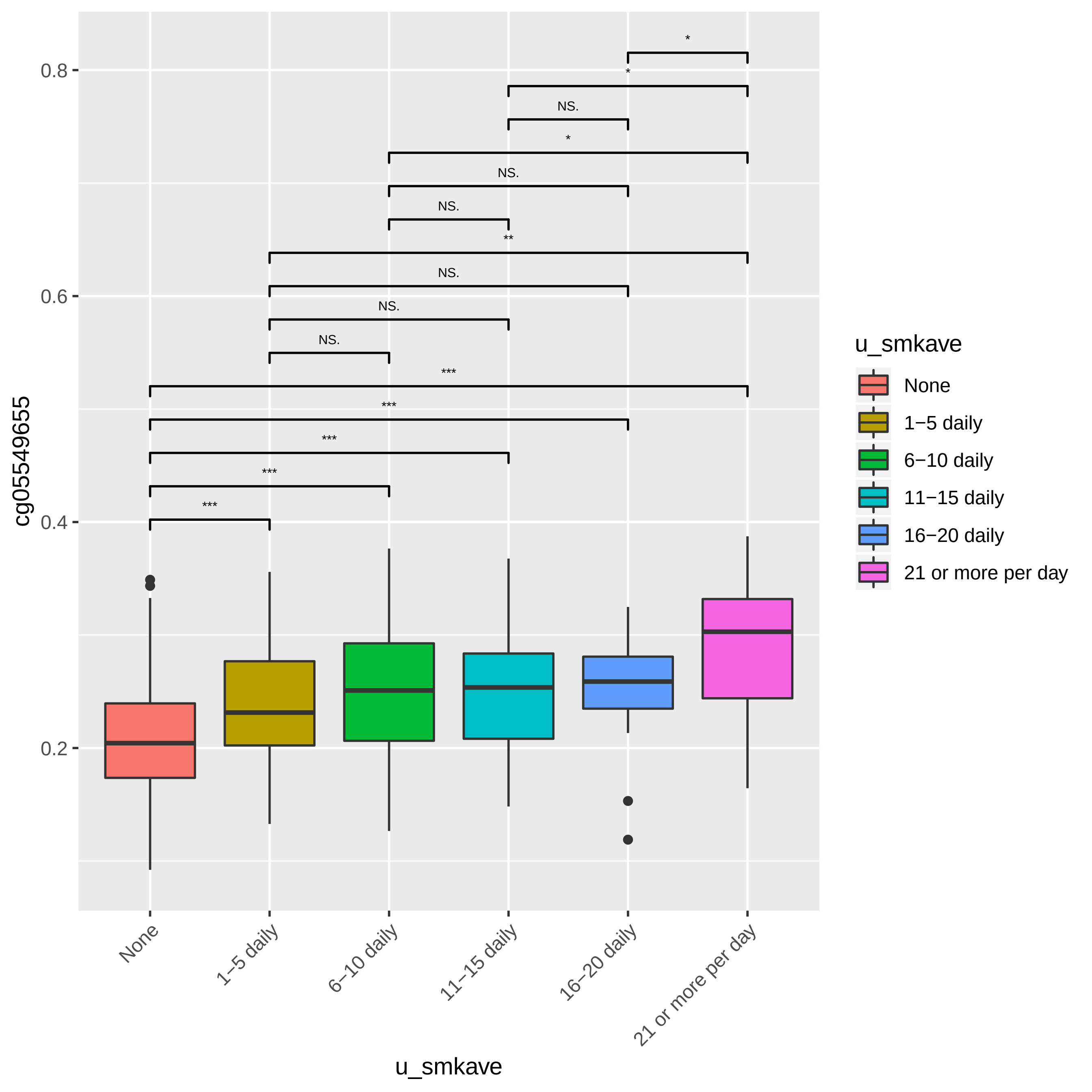

0.8
0.6
0.4
0.2
cg05549655
*
*
NS.
*
NS.
NS.
**
NS.
NS.
NS.
***
***
***
***
***
None
1−5 daily
6−10 daily
11−15 daily
16−20 daily
21 or more per day
u_smkave
u_smkave
None
1−5 daily
6−10 daily
11−15 daily
16−20 daily
21 or more per day

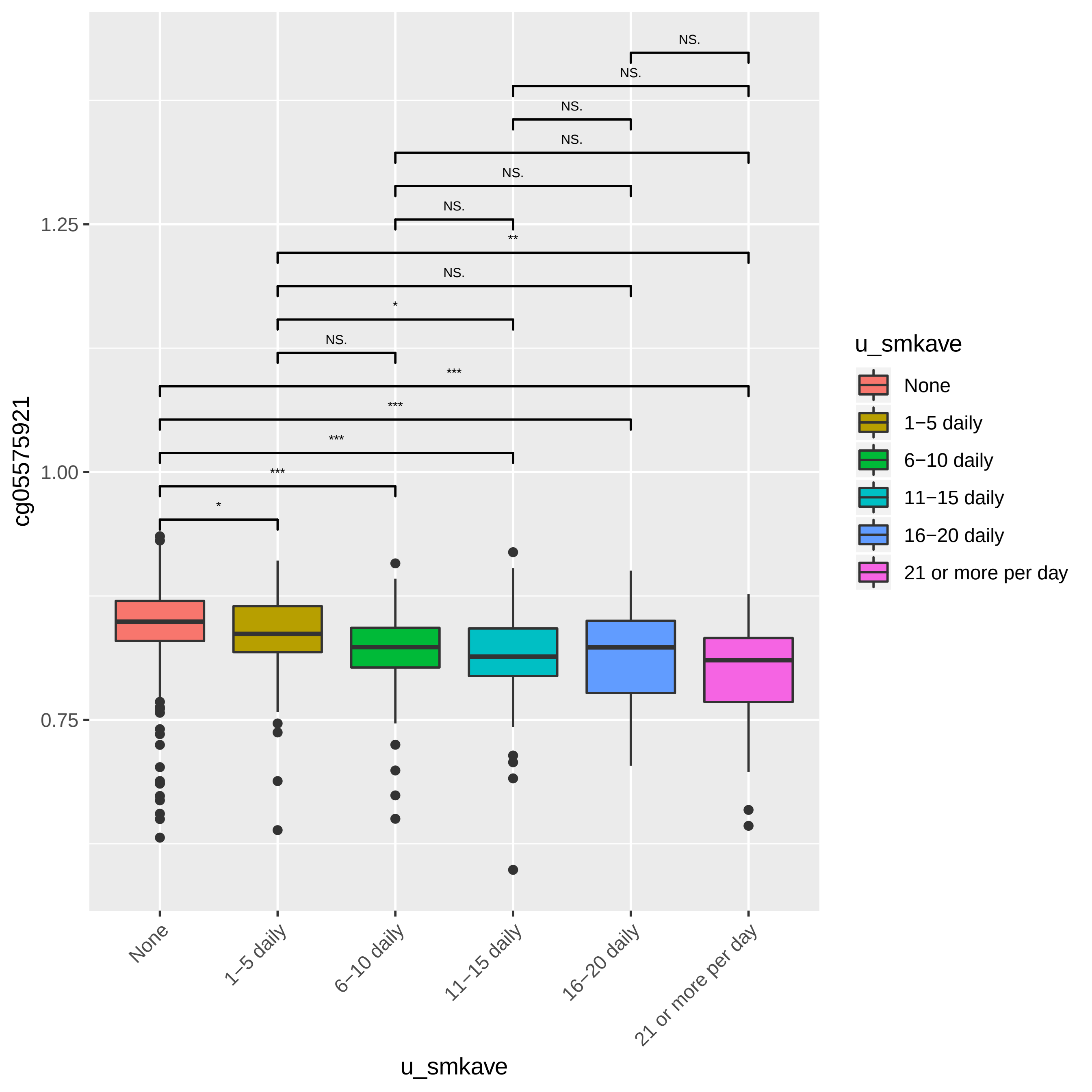

NS.
NS.
NS.
NS.
NS.
NS.
**
NS.
*
NS.
***
***
***
***
*
1.25
1.00
0.75
cg05575921
None
1−5 daily
6−10 daily
11−15 daily
16−20 daily
21 or more per day
u_smkave
u_smkave
None
1−5 daily
6−10 daily
11−15 daily
16−20 daily
21 or more per day

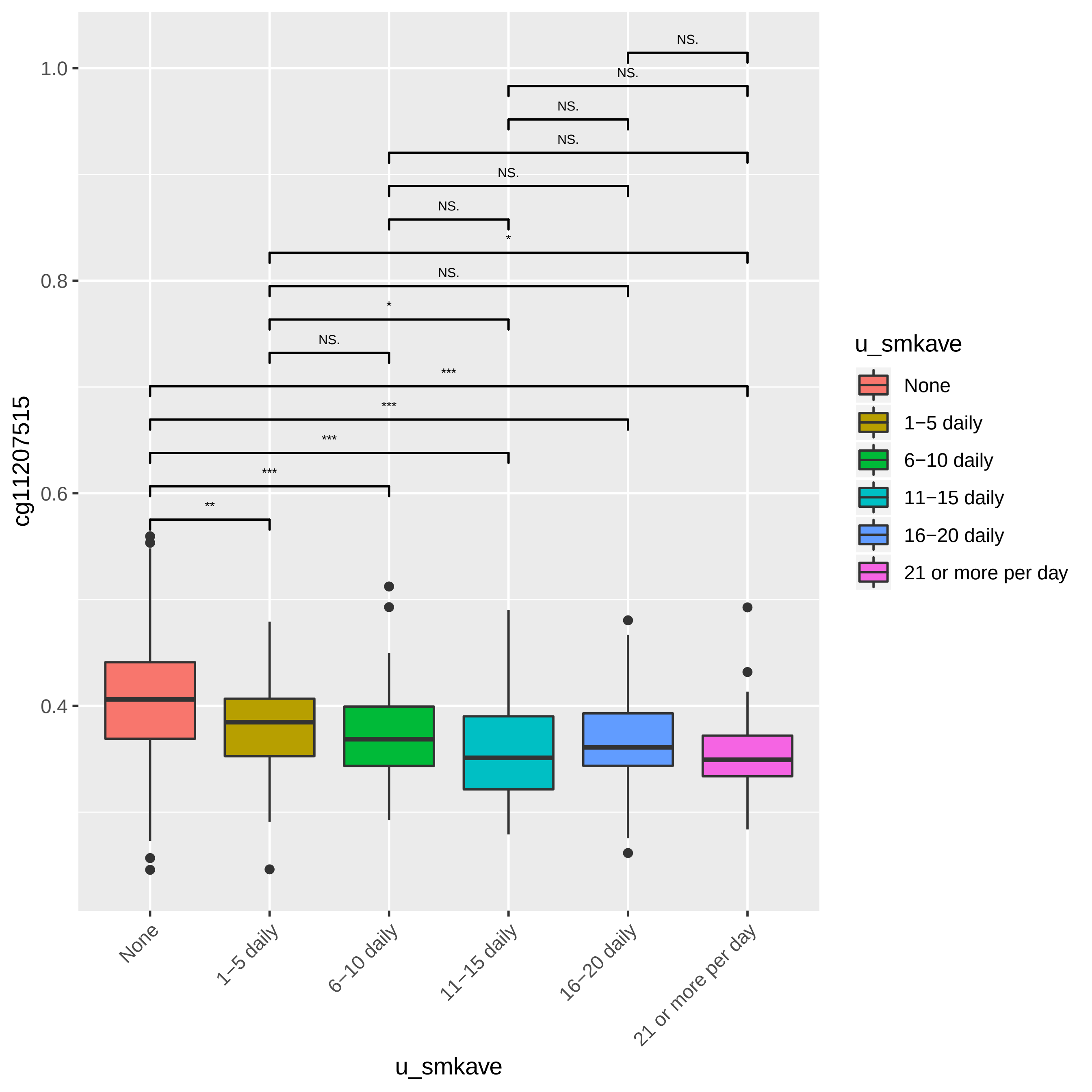

NS.
NS.
NS.
NS.
NS.
NS.
*
NS.
*
NS.
***
***
***
***
**
cg11207515
1.0
0.8
0.6
0.4
None
1−5 daily
6−10 daily
11−15 daily
16−20 daily
21 or more per day
u_smkave
u_smkave
None
1−5 daily
6−10 daily
11−15 daily
16−20 daily
21 or more per day

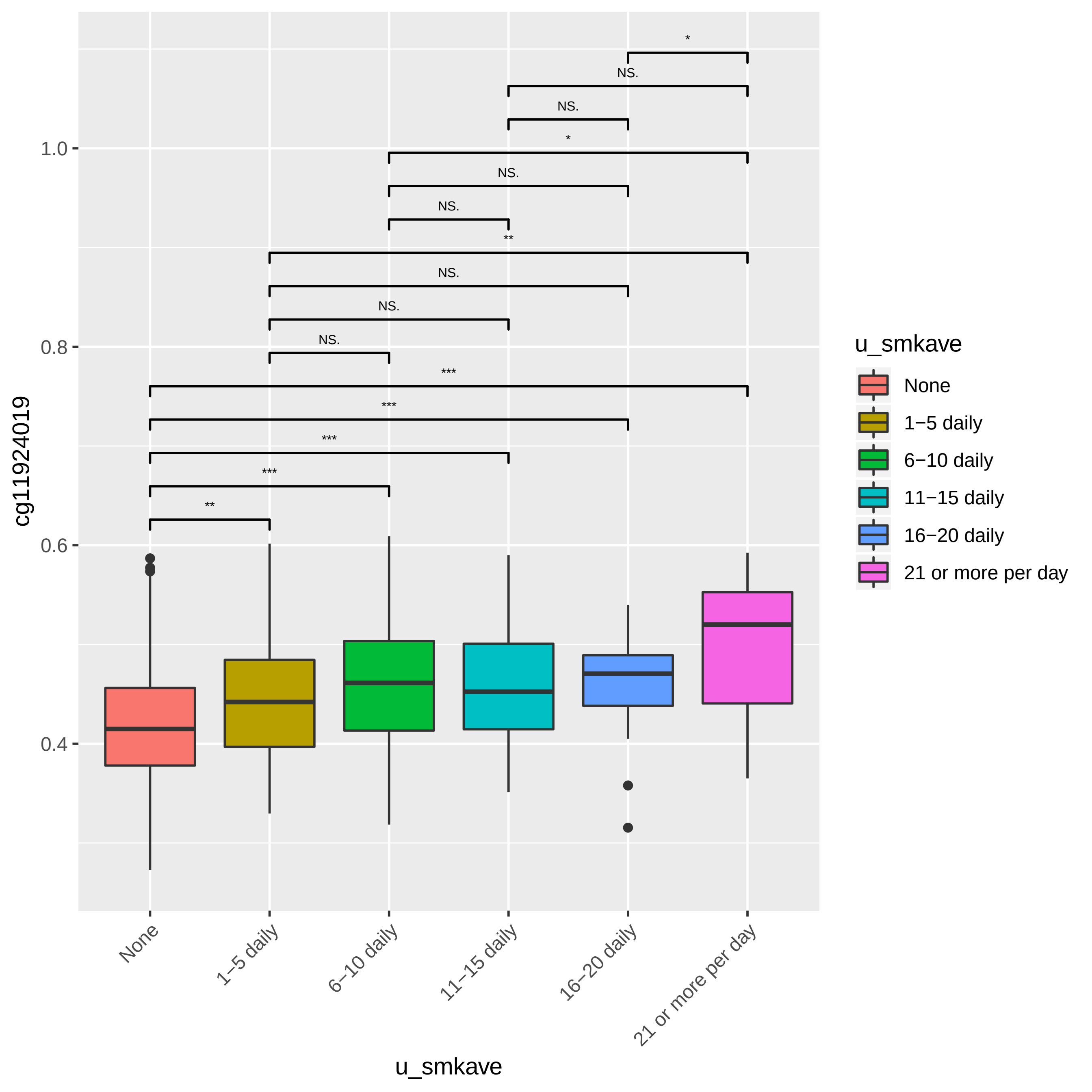

cg11924019
u_smkave
1.0
0.8
0.6
0.4
None
1−5 daily
6−10 daily
11−15 daily
16−20 daily
21 or more per day
*
NS.
NS.
*
NS.
NS.
**
NS.
NS.
NS.
***
***
***
***
**
u_smkave
None
1−5 daily
6−10 daily
11−15 daily
16−20 daily
21 or more per day

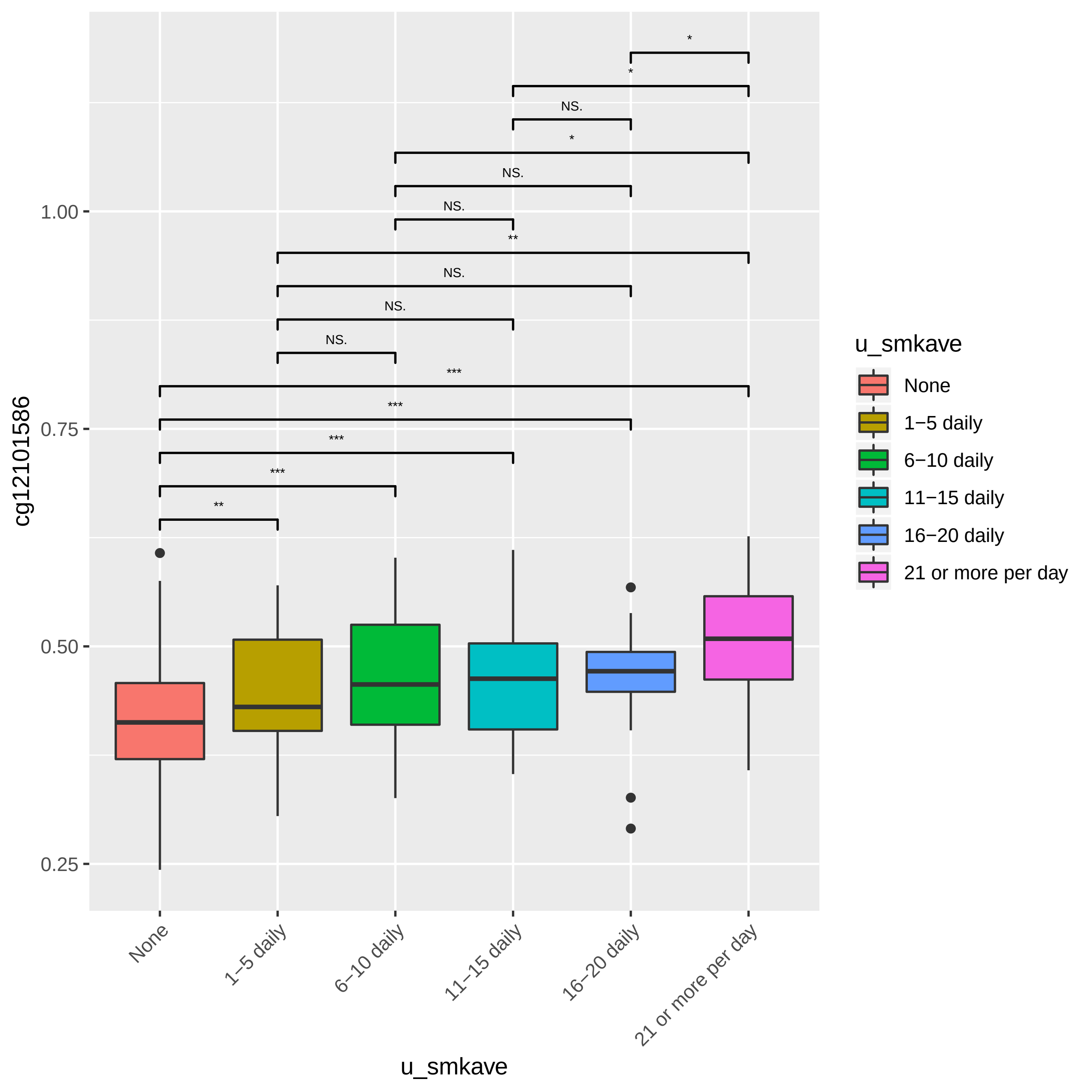

cg12101586
u_smkave
1.00
0.75
0.50
0.25
None
1−5 daily
6−10 daily
11−15 daily
16−20 daily
21 or more per day
**
***
***
***
***
NS.
NS.
NS.
**
NS.
NS.
*
NS.
*
*
u_smkave
None
1−5 daily
6−10 daily
11−15 daily
16−20 daily
21 or more per day

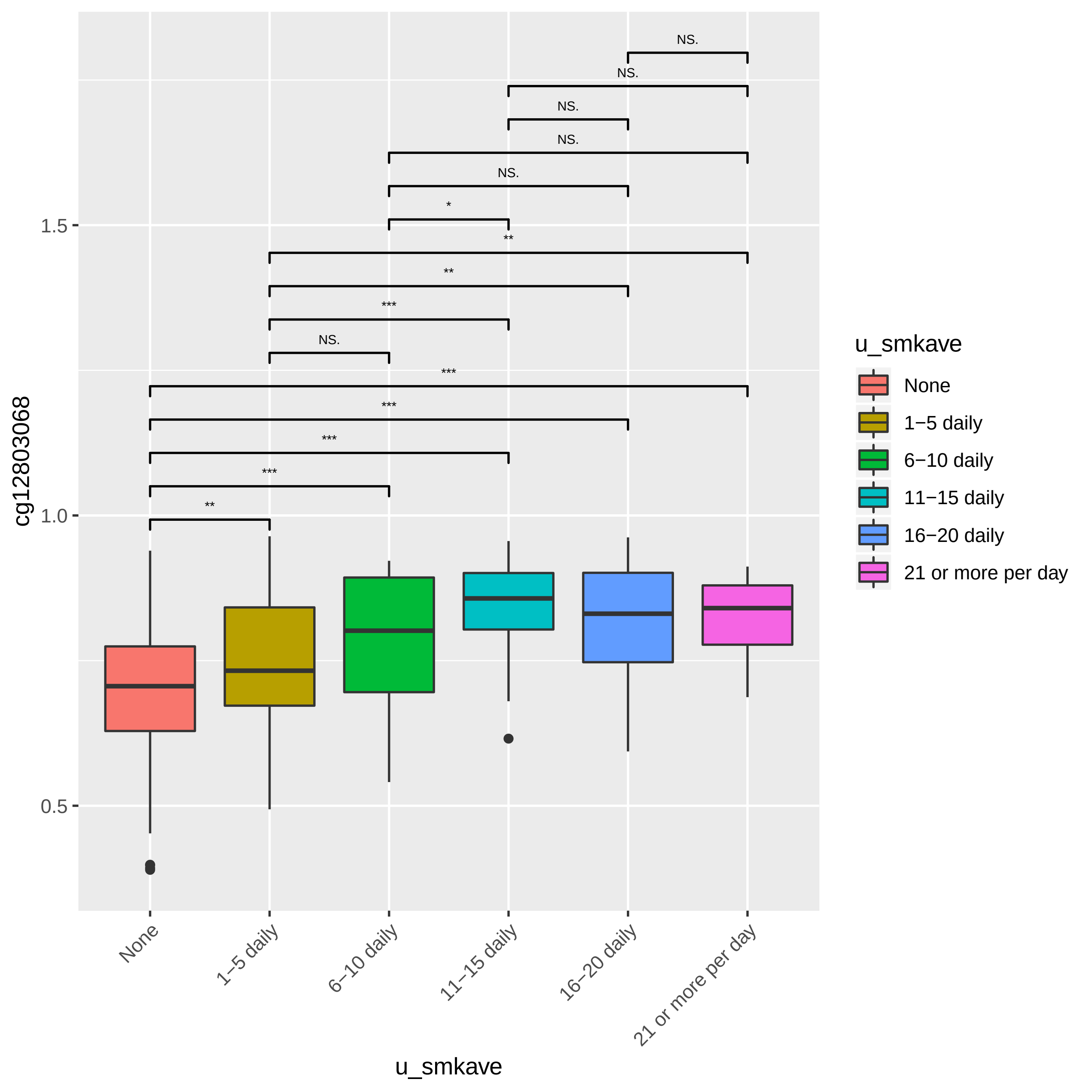

cg12803068
u_smkave
1.5
1.0
0.5
None
1−5 daily
6−10 daily
11−15 daily
16−20 daily
21 or more per day
NS.
*
**
***

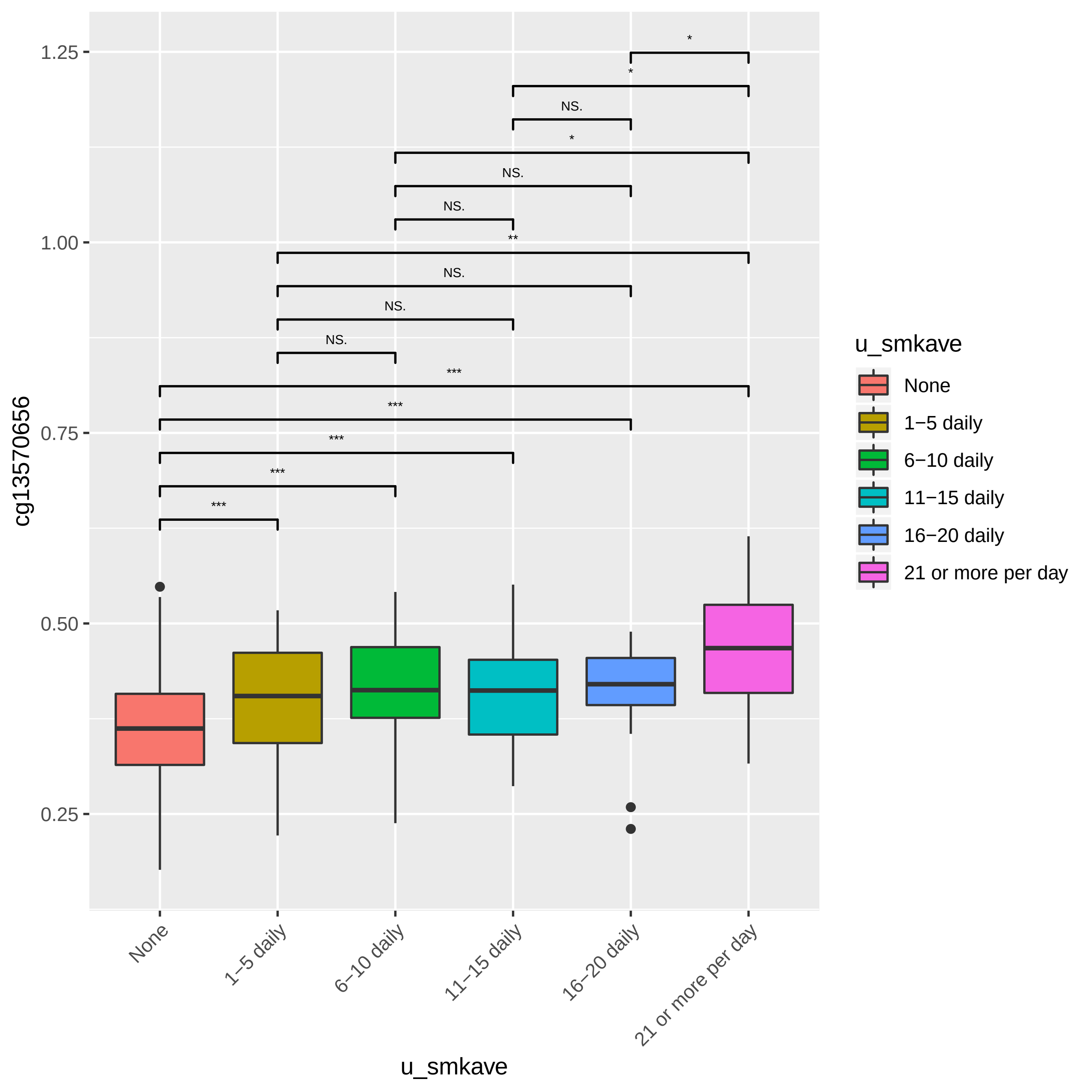
cg13570656
1.25
1.00
0.75
0.50
0.25
*
*
NS.
*
NS.
NS.
**
NS.
NS.
NS.
***
***
***
***
***
None
1−5 daily
6−10 daily
11−15 daily
16−20 daily
21 or more per day
u_smkave
u_smkave
None
1−5 daily
6−10 daily
11−15 daily
16−20 daily
21 or more per day

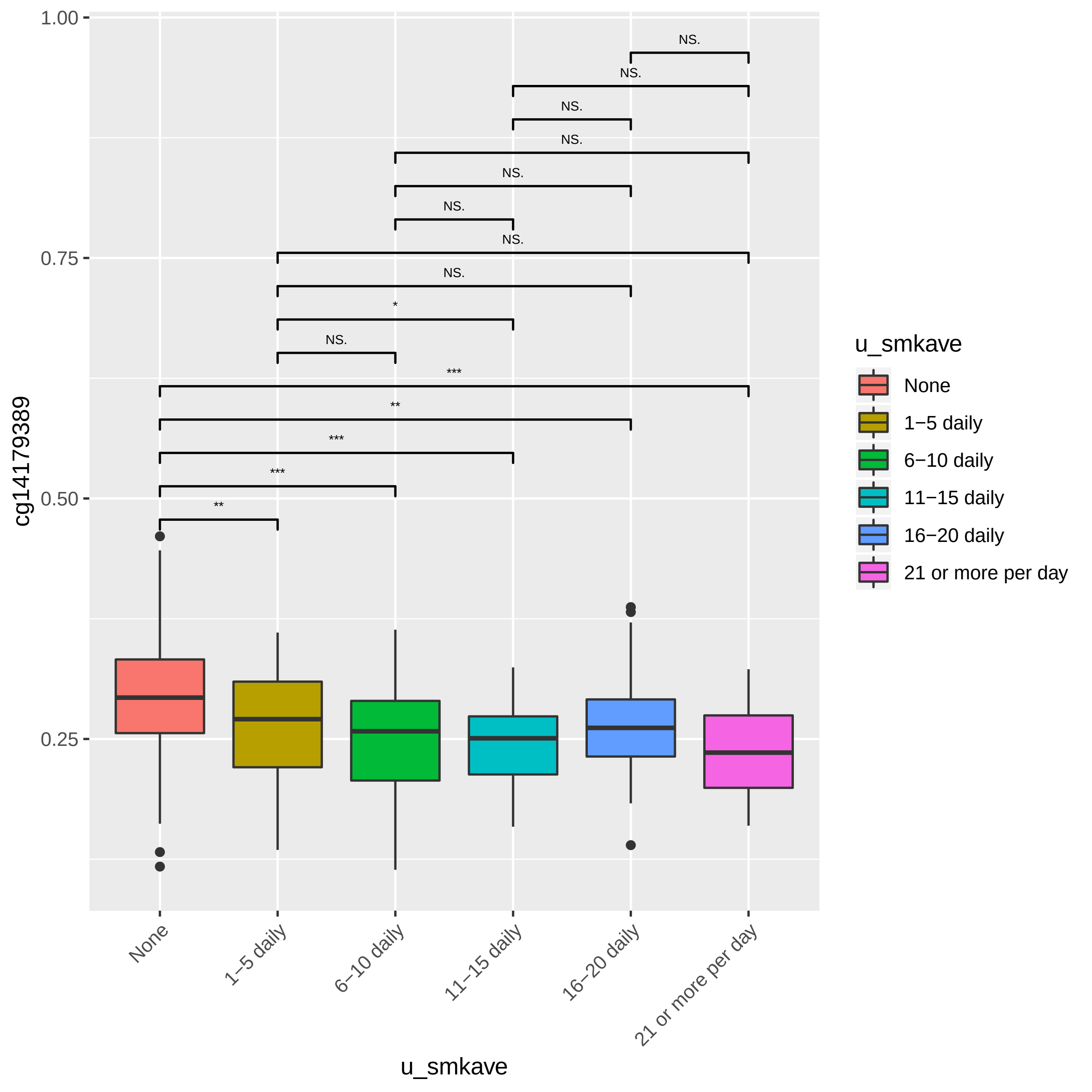

1.00
0.75
0.50
0.25
cg14179389
NS.
NS.
NS.
NS.
NS.
NS.
NS.
NS.
*
NS.
***
**
***
***
**
None
1−5 daily
6−10 daily
11−15 daily
16−20 daily
21 or more per day
u_smkave
u_smkave
None
1−5 daily
6−10 daily
11−15 daily
16−20 daily
21 or more per day

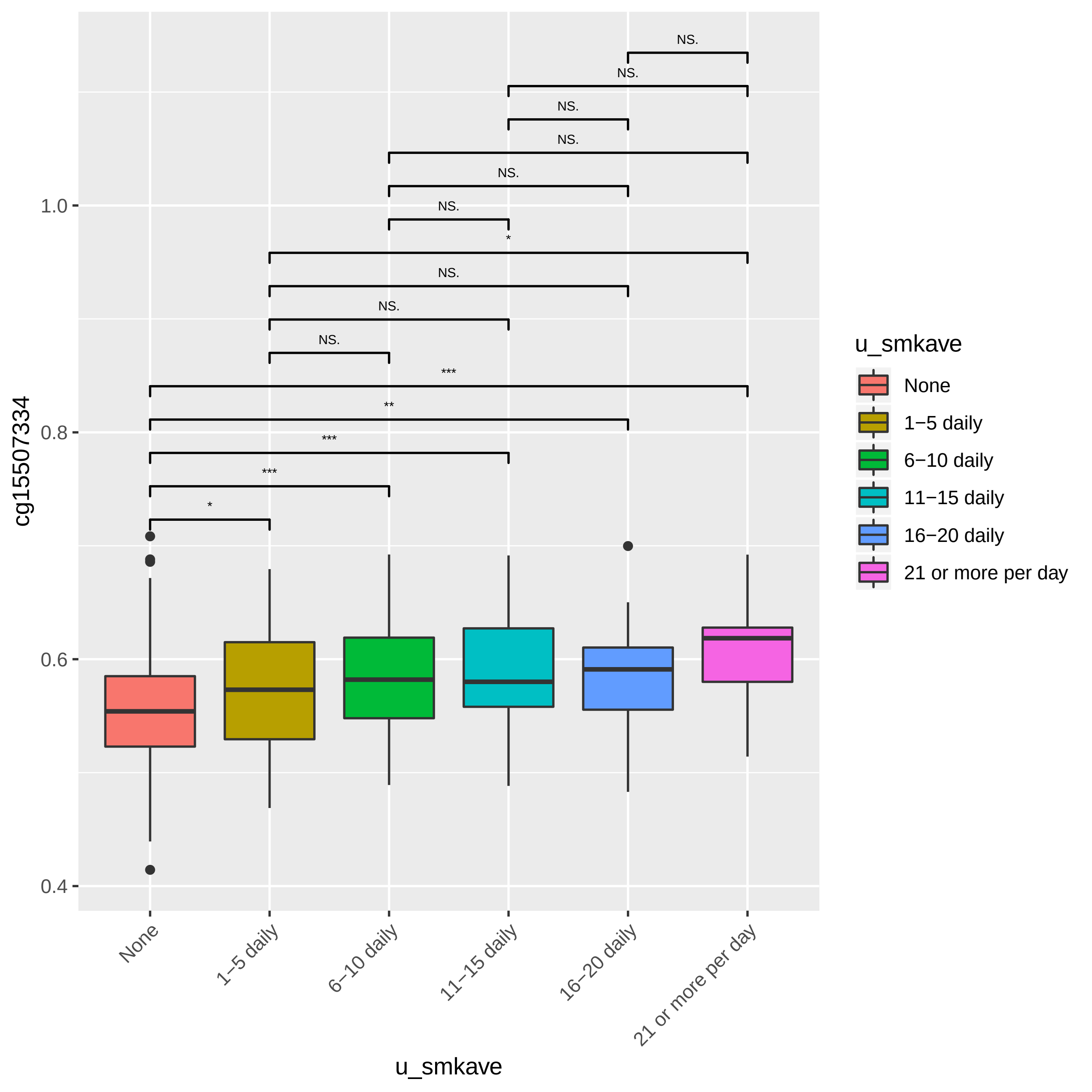
NS.
NS.
NS.
NS.
NS.
NS.
*
NS.
NS.
NS.
***
**
***
***
*
1.0
0.8
0.6
0.4
cg15507334
None
1−5 daily
6−10 daily
11−15 daily
16−20 daily
21 or more per day
u_smkave
u_smkave
None
1−5 daily
6−10 daily
11−15 daily
16−20 daily
21 or more per day

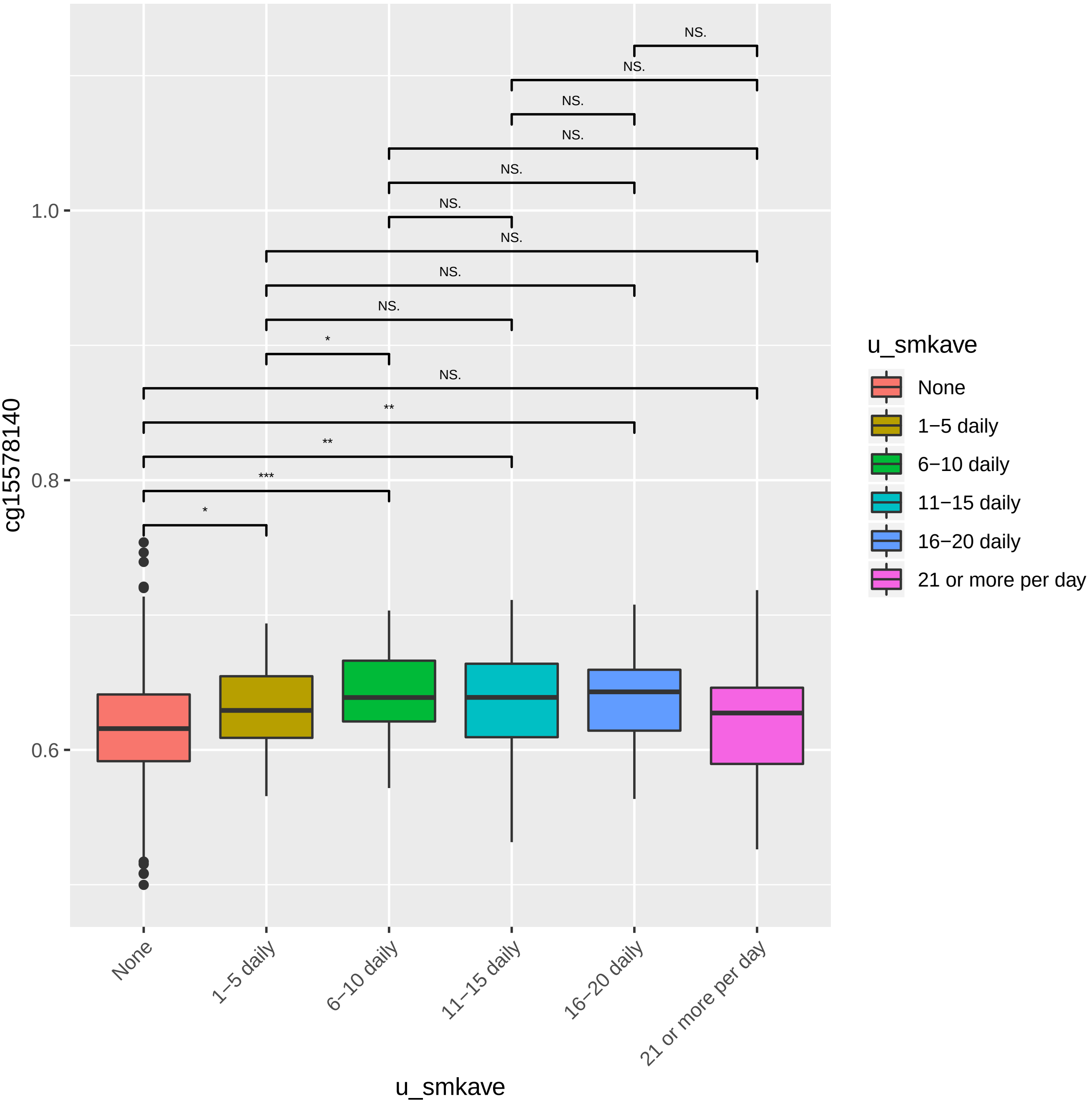
NS.
NS.
NS.
NS.
NS.
NS.
NS.
NS.
NS.
*
NS.
**
**
***
*
1.0
0.8
0.6
cg15578140
None
1−5 daily
6−10 daily
11−15 daily
16−20 daily
21 or more per day
u_smkave
u_smkave
None
1−5 daily
6−10 daily
11−15 daily
16−20 daily
21 or more per day

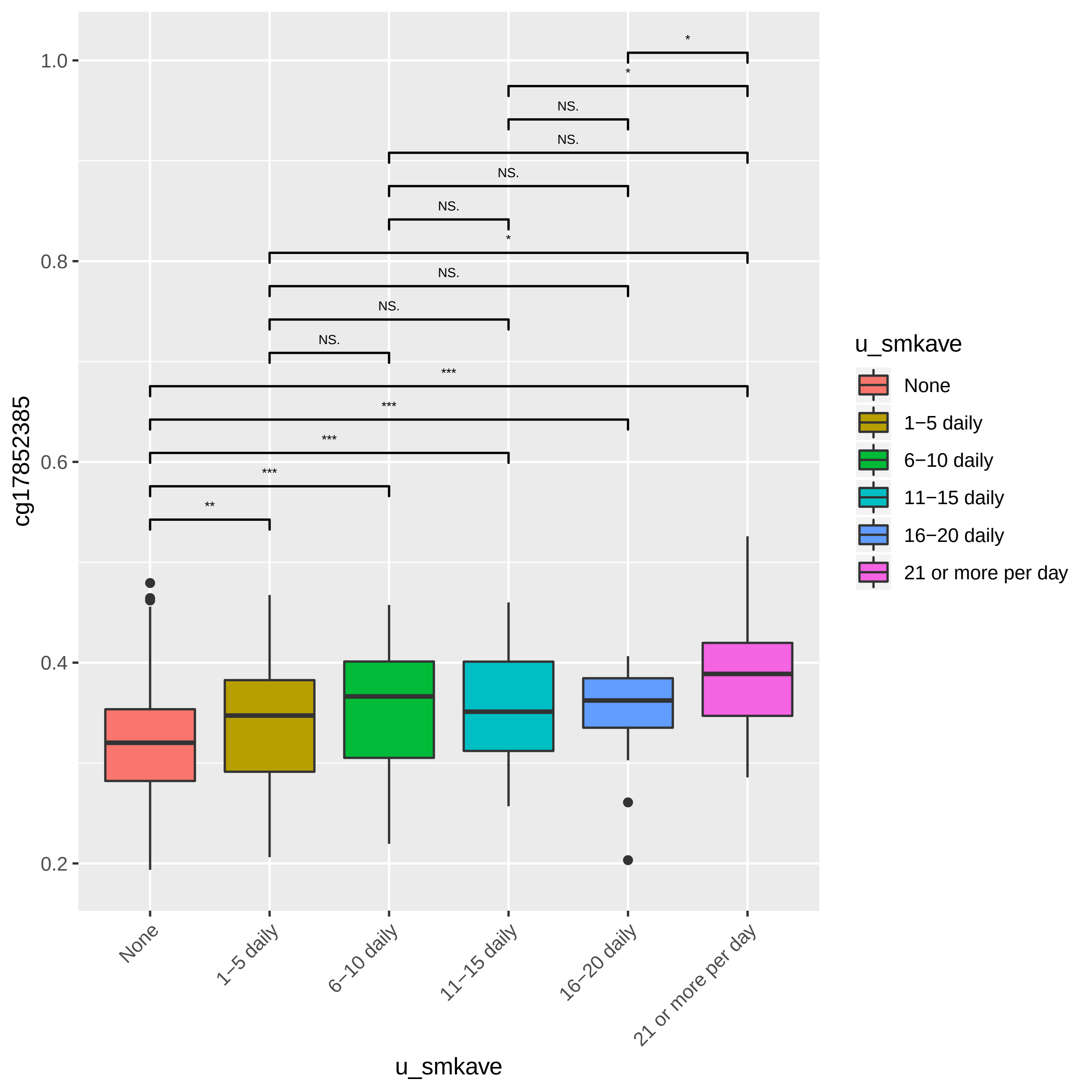

cg17852385
u_smkave
None
1−5 daily
6−10 daily
11−15 daily
16−20 daily
21 or more per day
0.2
0.4
0.6
0.8
1.0
NS.
*
**
***

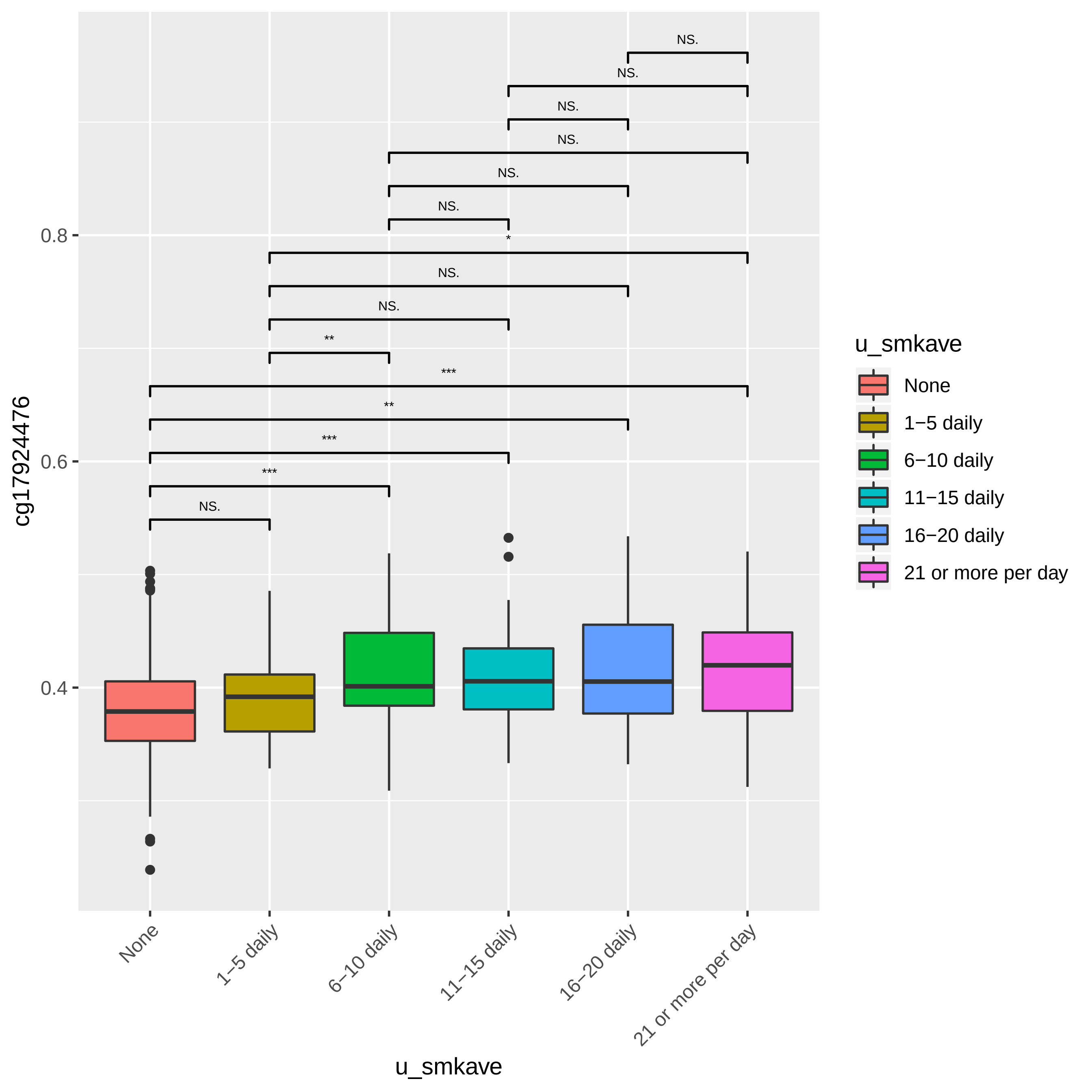

NS.
NS.
NS.
NS.
NS.
NS.
*
NS.
NS.
**
***
**
***
***
NS.
cg17924476
0.8
0.6
0.4
None
1−5 daily
6−10 daily
11−15 daily
16−20 daily
21 or more per day
u_smkave
u_smkave
None
1−5 daily
6−10 daily
11−15 daily
16−20 daily
21 or more per day

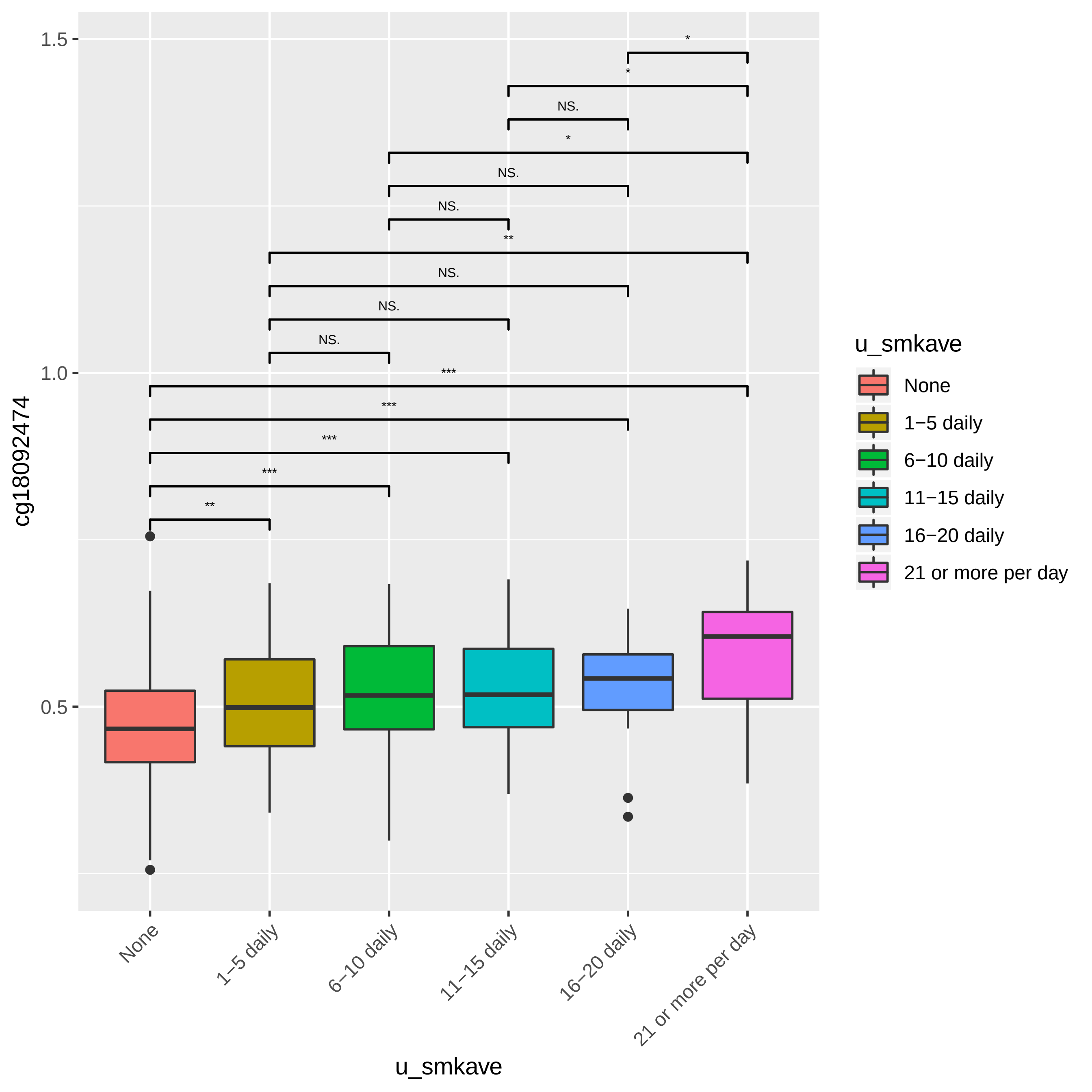
u_smkave
None
1−5 daily
6−10 daily
11−15 daily
16−20 daily
21 or more per day
cg18092474
1.5
1.0
0.5
*
NS.
**
***

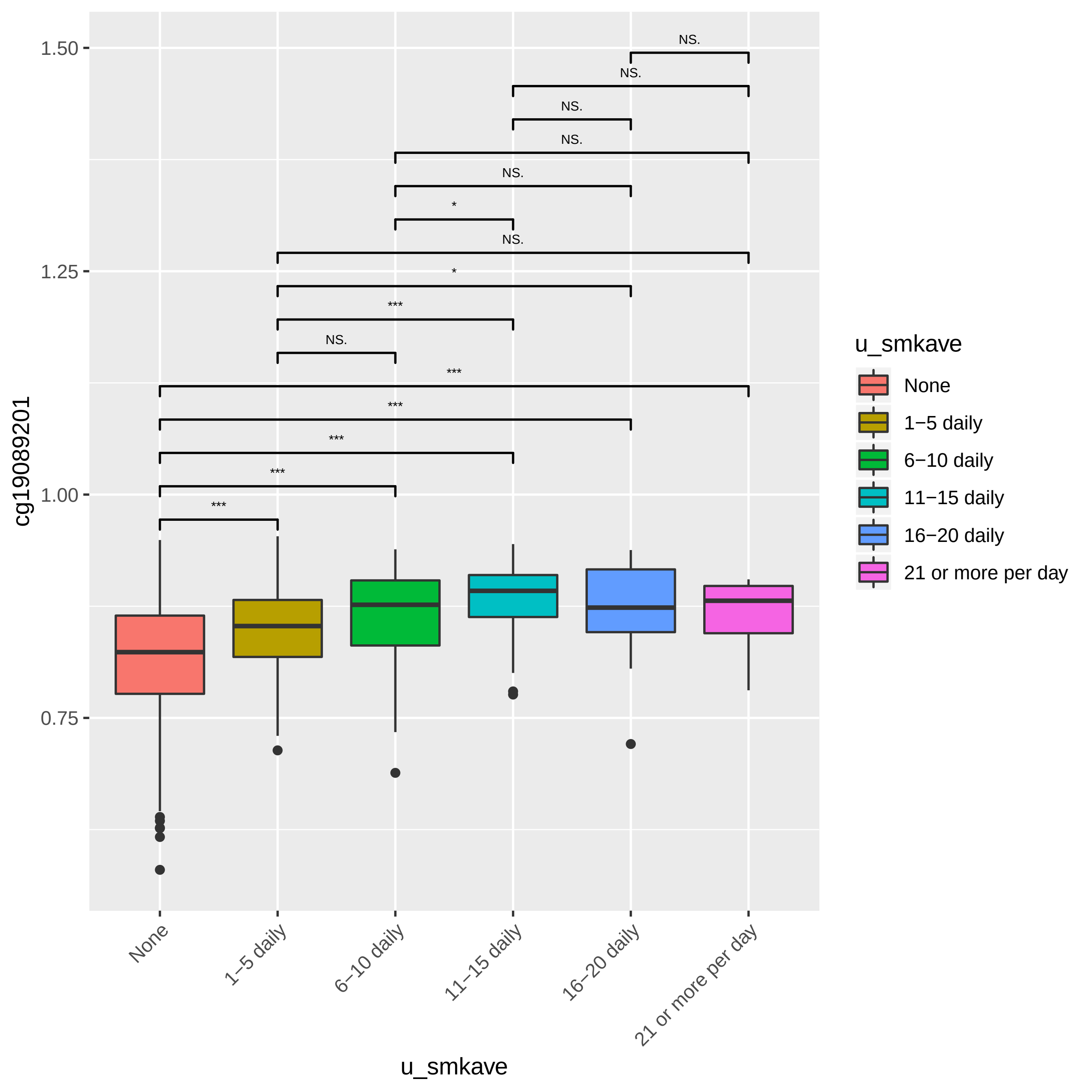

cg19089201
1.50
1.25
1.00
0.75
NS.
*
***
None
1−5 daily
6−10 daily
11−15 daily
16−20 daily
21 or more per day
u_smkave

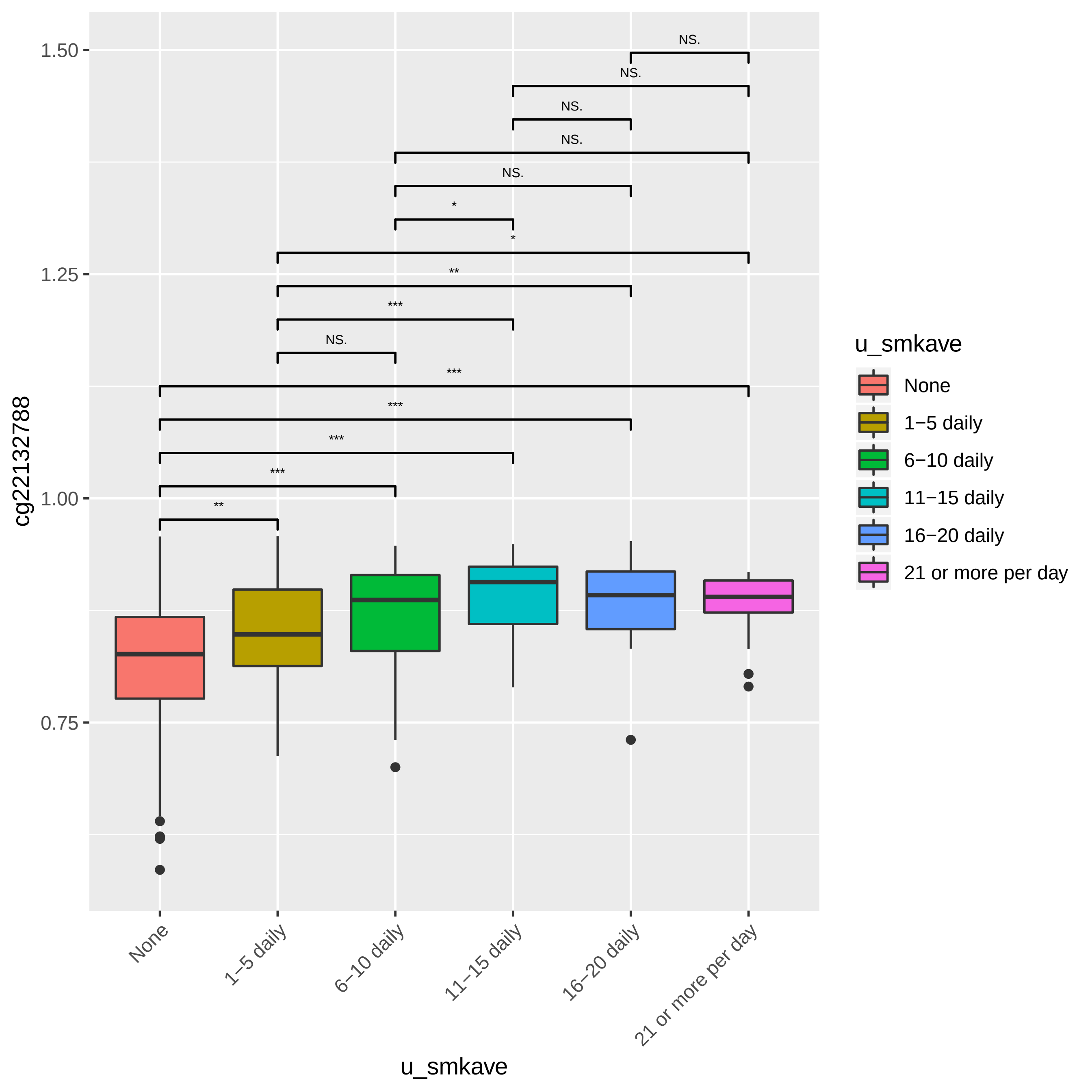

cg22132788
1.50
1.25
1.00
0.75
u_smkave
None
1−5 daily
6−10 daily
11−15 daily
16−20 daily
21 or more per day
NS.
*
**
***

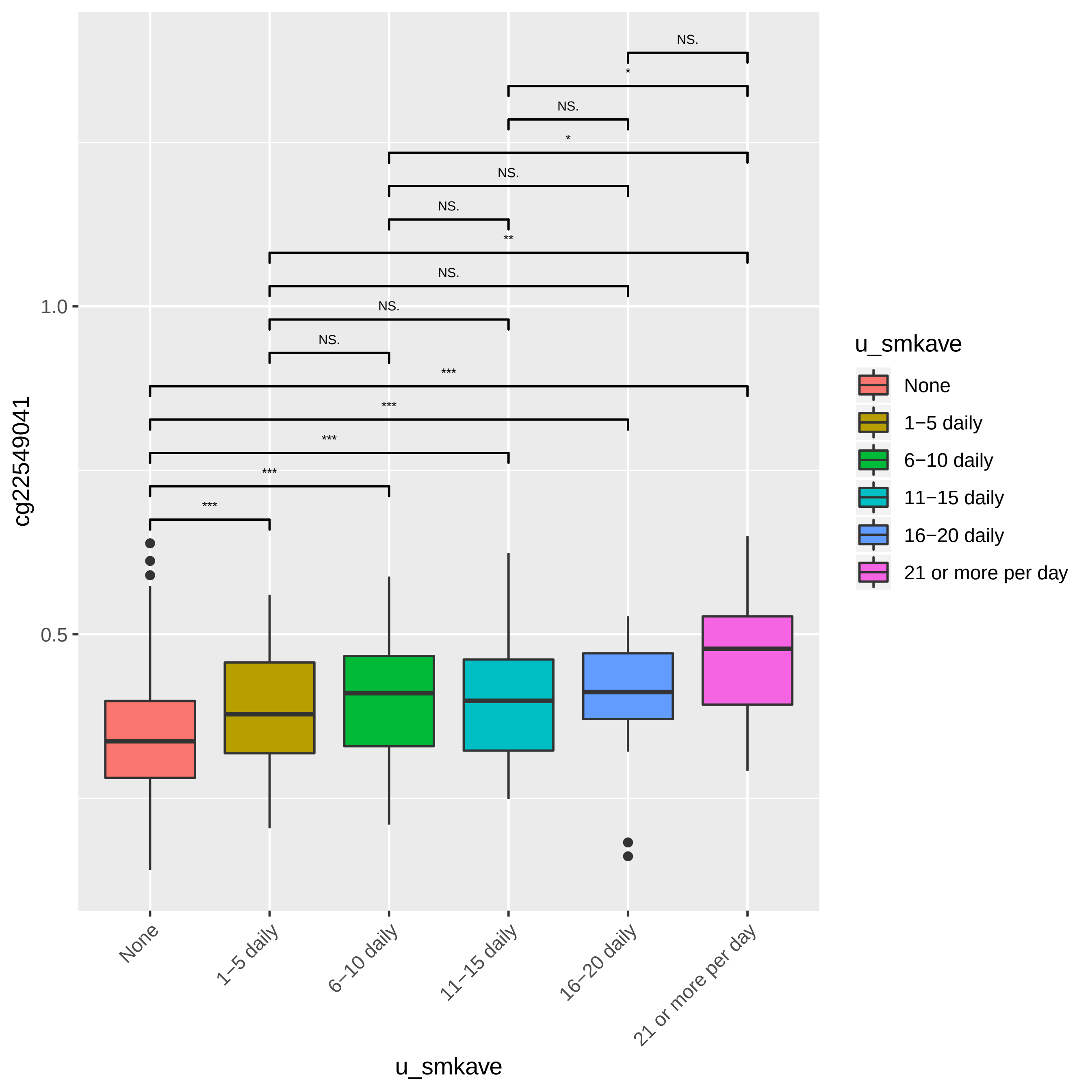
NS.
*
NS.
*
NS.
NS.
**
NS.
NS.
NS.
***
***
***
***
***
1.0
0.5
cg22549041
u_smkave
None
1−5 daily
6−10 daily
11−15 daily
16−20 daily
21 or more per day
u_smkave
None
1−5 daily
6−10 daily
11−15 daily
16−20 daily
21 or more per day

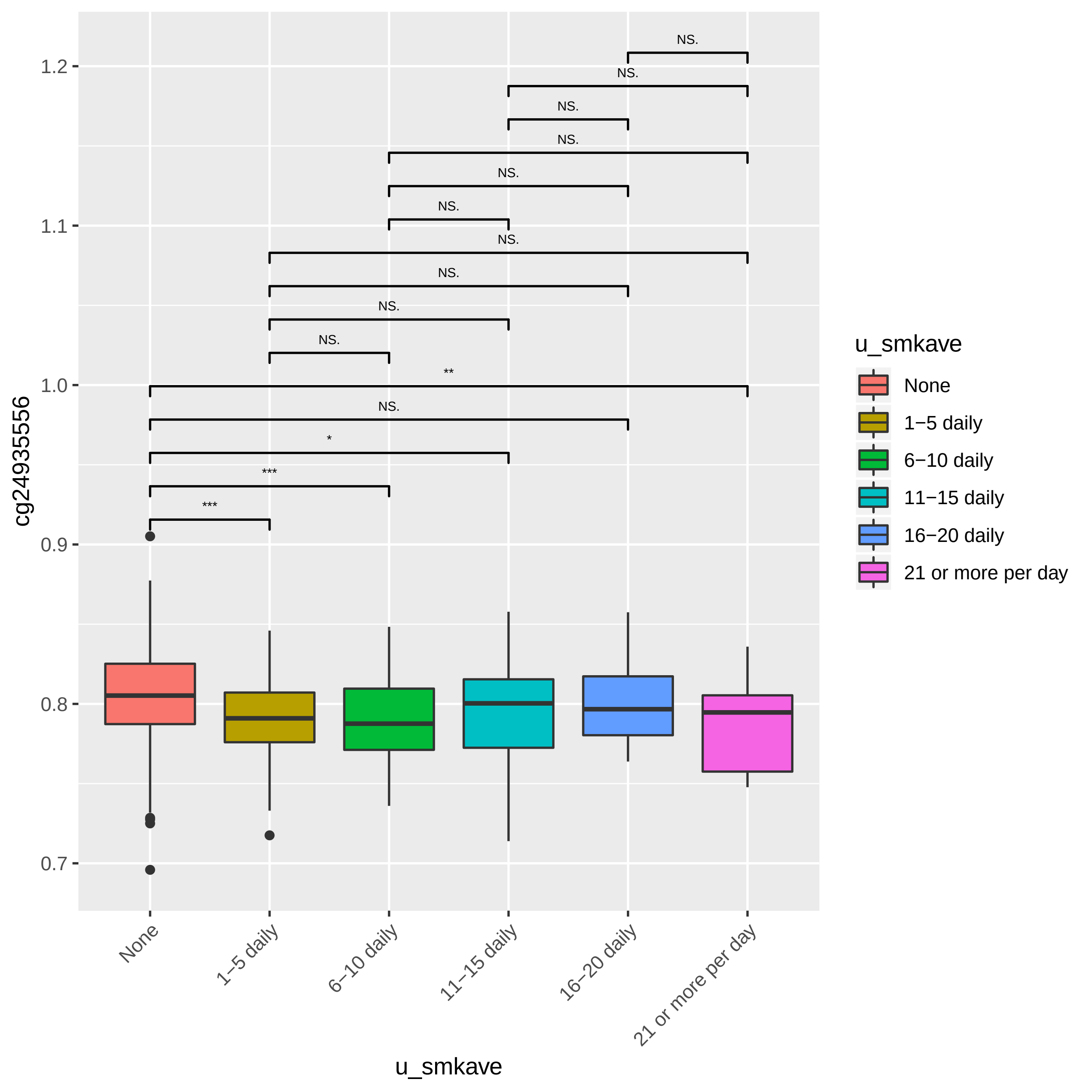

NS.
NS.
NS.
NS.
NS.
NS.
NS.
NS.
NS.
NS.
**
NS.
*
***
***
1.2
1.1
1.0
0.9
0.8
0.7
cg24935556
None
1−5 daily
6−10 daily
11−15 daily
16−20 daily
21 or more per day
u_smkave
u_smkave
None
1−5 daily
6−10 daily
11−15 daily
16−20 daily
21 or more per day

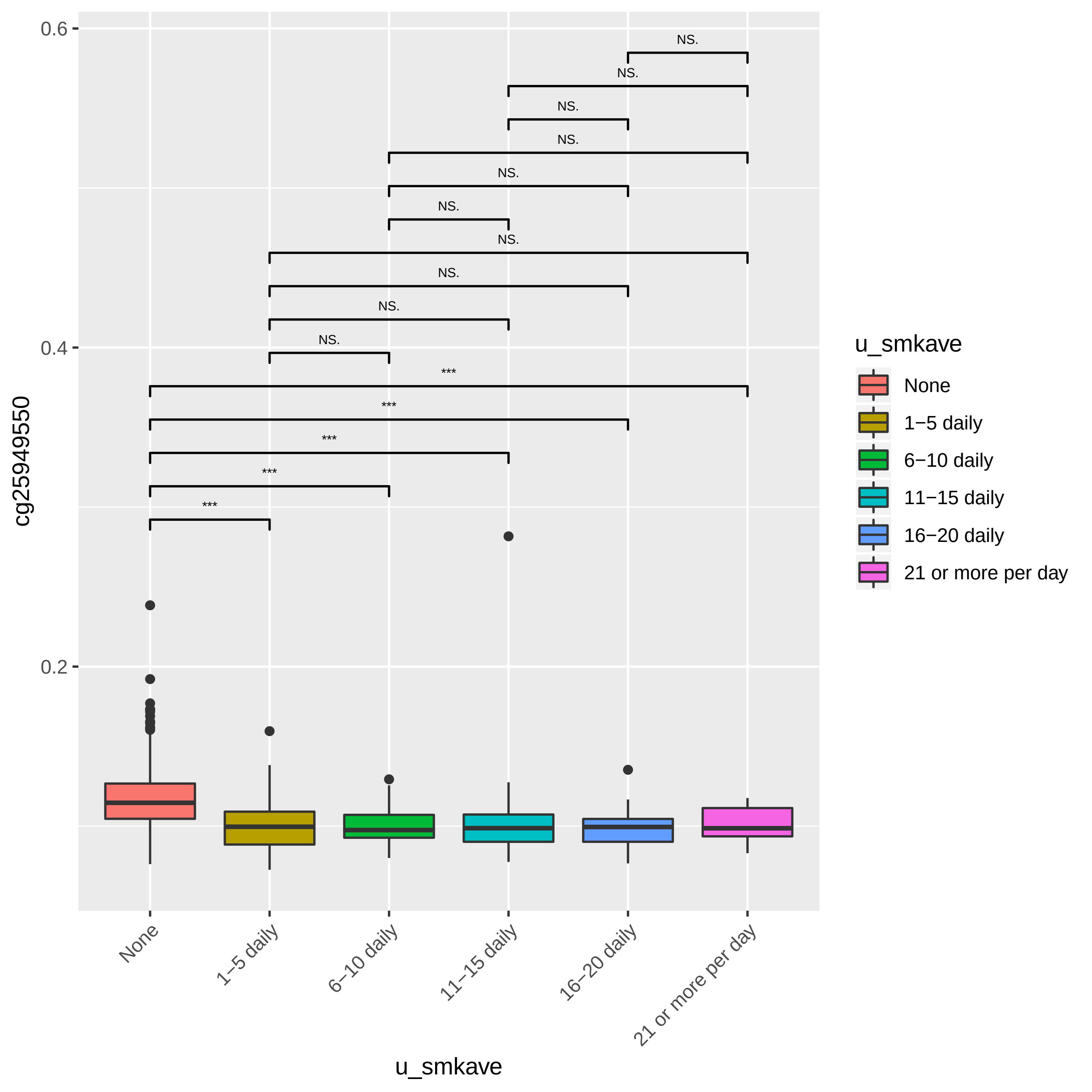
0.6
0.4
0.2
cg25949550
NS.
***
None
1−5 daily
6−10 daily
11−15 daily
16−20 daily
21 or more per day
u_smkave
u_smkave
None
1−5 daily
6−10 daily
11−15 daily
16−20 daily
21 or more per day

Supplemental Figure S2. Comparison of 95% confidence interval and beta coefficient of the regression model with the CpGs on the y-axis as outcome and the smoking variables in the legend as predictor, adjusted for sex, offspring age, age of the mother, birthweight, gestational weight gain, maternal alcohol consumption during pregnancy, maternal school level, maternal pre-pregnancy BMI, family income during pregnancy, cell count and batch effects.

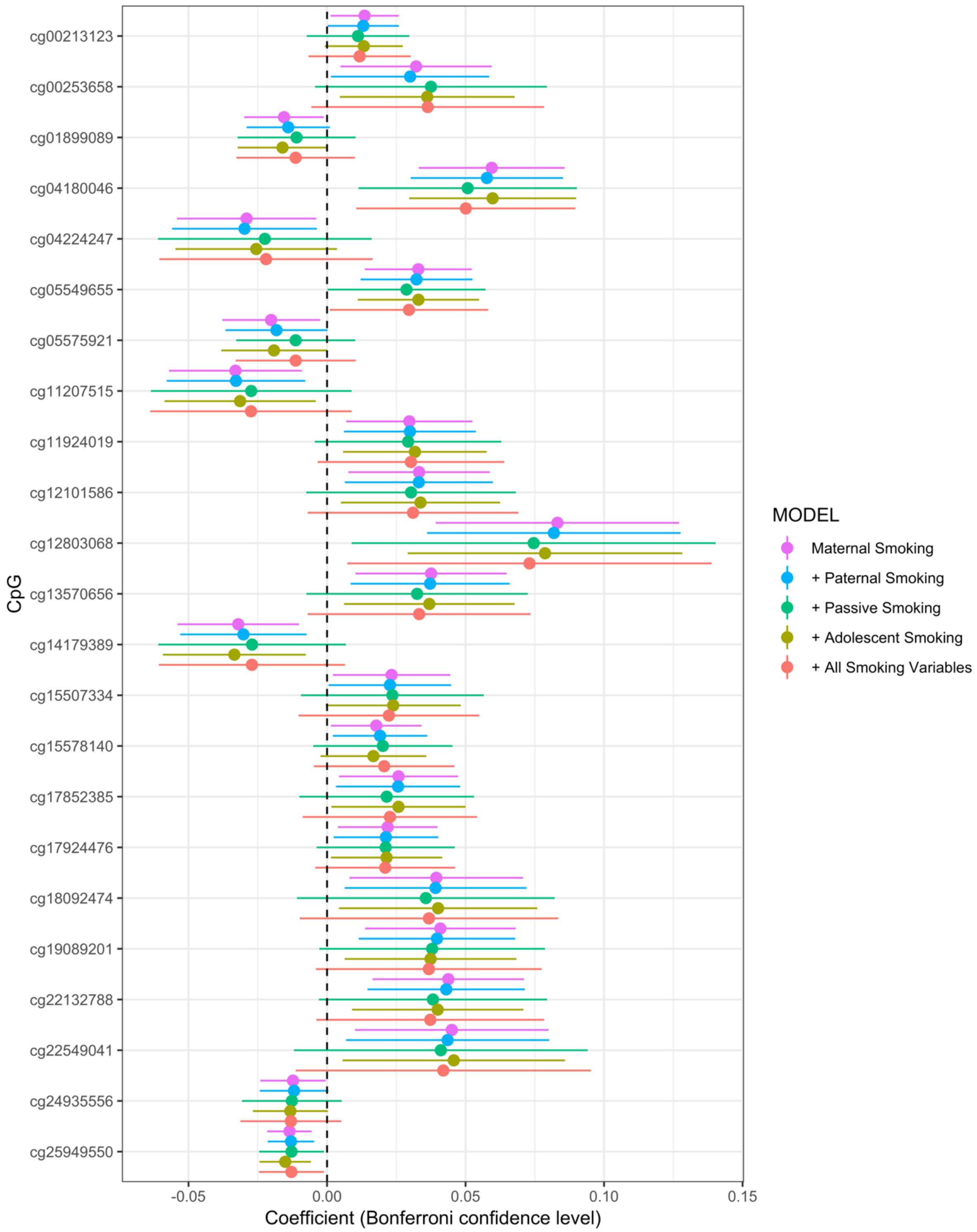

cg00213123
cg00253658
cg01899089
cg04180046
cg04224247
cg05549655
cg05575921
cg11207515
cg11924019
cg12101586
cg12803068
cg13570656
cg14179389
cg15507334
cg15578140
cg17852385
cg17924476
cg18092474
cg19089201
cg22132788
cg22549041
cg24935556
cg25949550
CpG
-0.05
0.00
0.05
0.10
0.15
Coefficient (Bonferroni confidence level)
MODEL
Maternal Smoking
+ Paternal Smoking
+ Passive Smoking
+ Adolescent Smoking
+ All Smoking Variables

Supplemental Figure S3. Coefficients and 95% confidence interval for the regression models with DNA methylation as outcome and maternal smoking during pregnancy as predictor, adjusted for sex, offspring age, age of the mother, birthweight, gestational weight gain, maternal alcohol consumption during pregnancy, maternal school level, maternal pre-pregnancy BMI, family income during pregnancy, cell count and batch effects.
Further, the model was stratified into a female and a male subset for comparison.

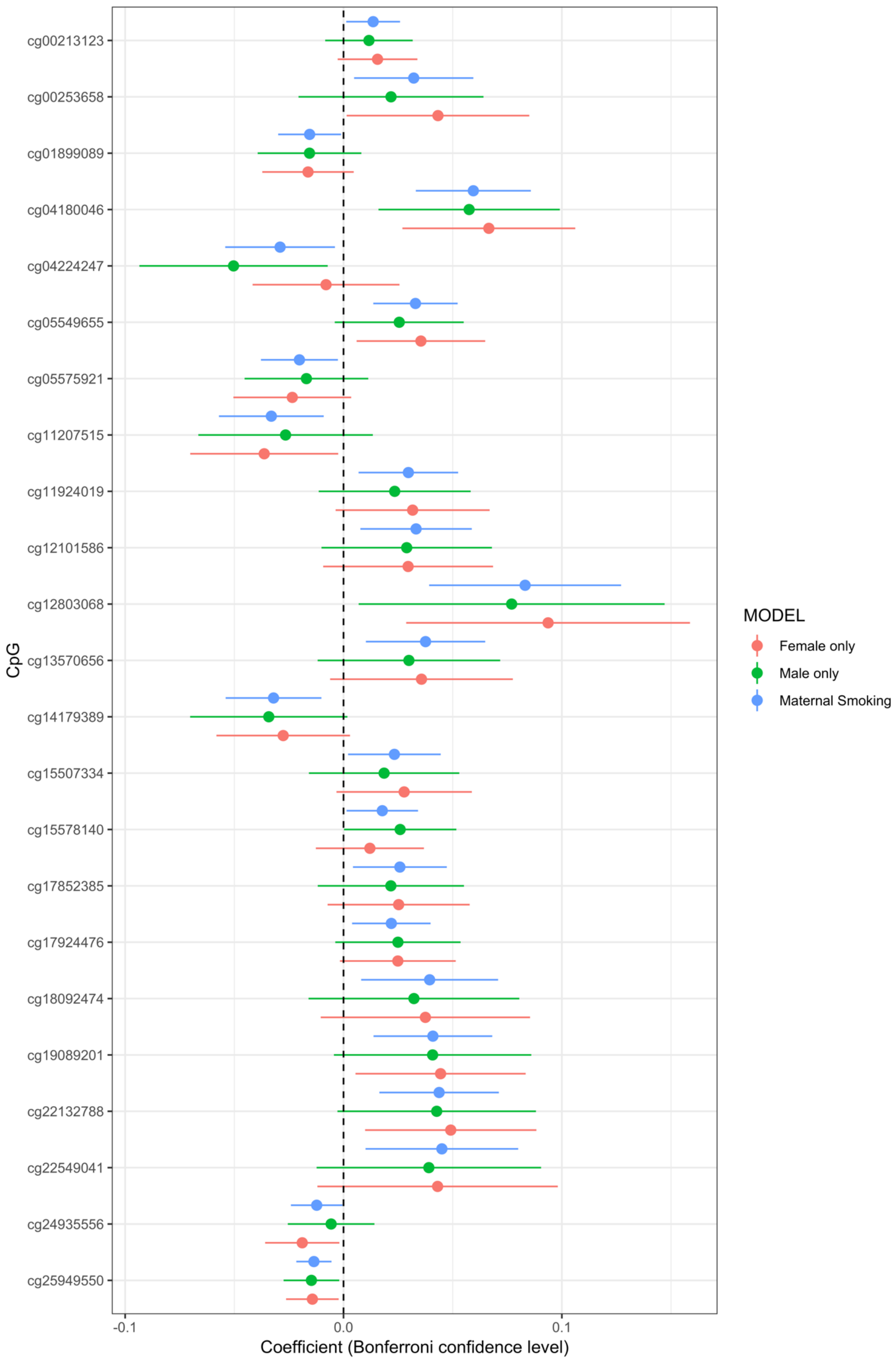

cg00213123
cg00253658
cg01899089
cg04180046
cg04224247
cg05549655
cg05575921
cg11207515
cg11924019
cg12101586
cg12803068
cg13570656
cg14179389
cg15507334
cg15578140
cg17852385
cg17924476
cg18092474
cg19089201
cg22132788
cg22549041
cg24935556
cg25949550
CpG
-0.1
0.0
0.1
Coefficient (Bonferroni confidence level)
MODEL
Female only
Male only
Maternal Smoking

Supplemental Figure S4. DNA methylation as outcome, different subsets of smoke exposure as categorical predictor, adjusted for sex, offspring age, age of the mother, birthweight, gestational weight gain, maternal alcohol consumption during pregnancy, maternal school level, maternal pre-pregnancy BMI, family income during pregnancy, cell count and batch effects.

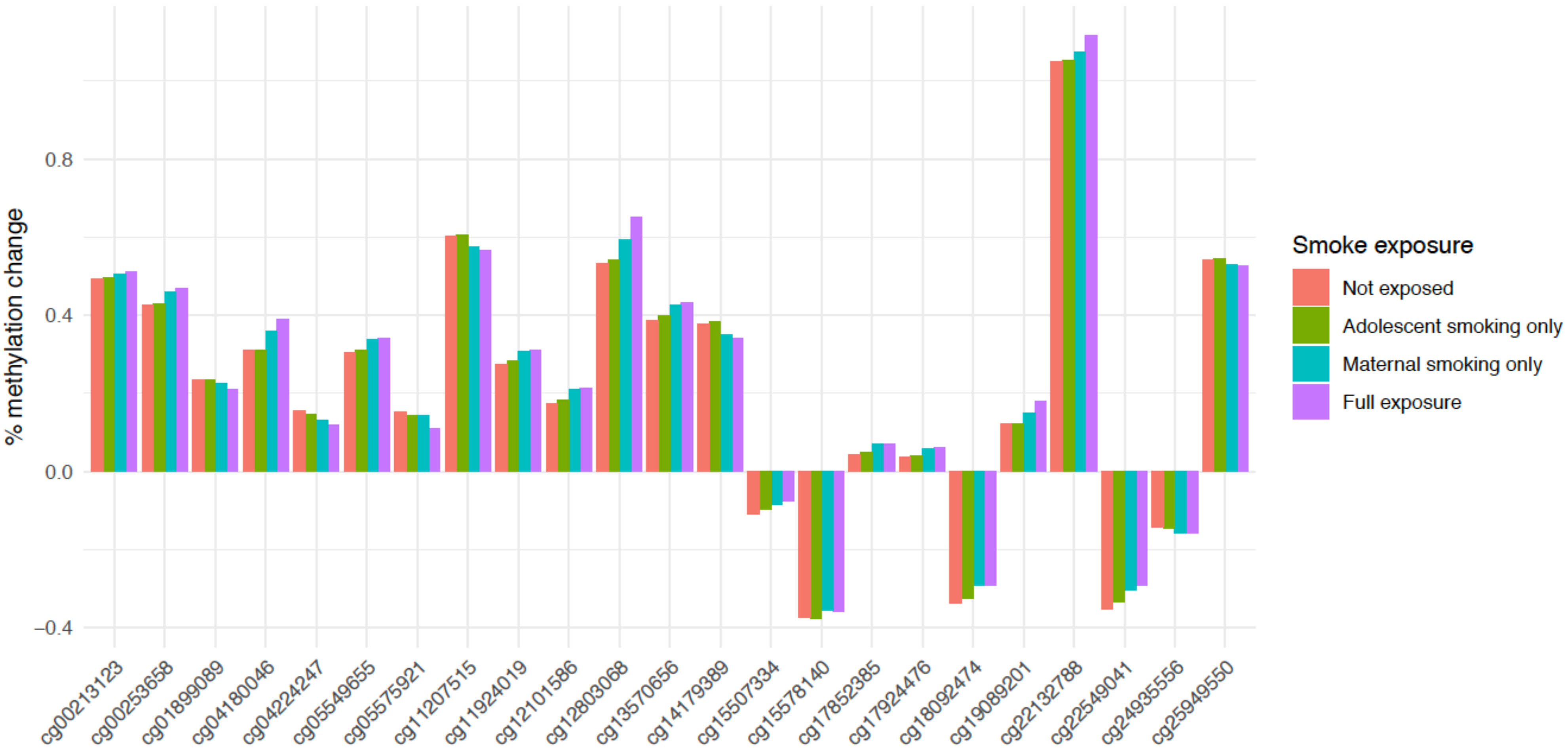

% methylation change
0.8
0.4
0.0
−0.4
cg00213123
cg00253658
cg01899089
cg04180046
cg04224247
cg05549655
cg05575921
cg11207515
cg11924019
cg12101586
cg12803068
cg13570656
cg14179389
cg15507334
cg15578140
cg17852385
cg17924476
cg18092474
cg19089201
cg22132788
cg22549041
cg24935556
cg25949550
Smoke exposure
Not exposed
Adolescent smoking only
Maternal smoking only
Full exposure

Supplemental Figure S5. Boxplot for BMI, waist circumference, triacylglycerol and HDL-cholesterol, stratified by in utero smoke exposure status.

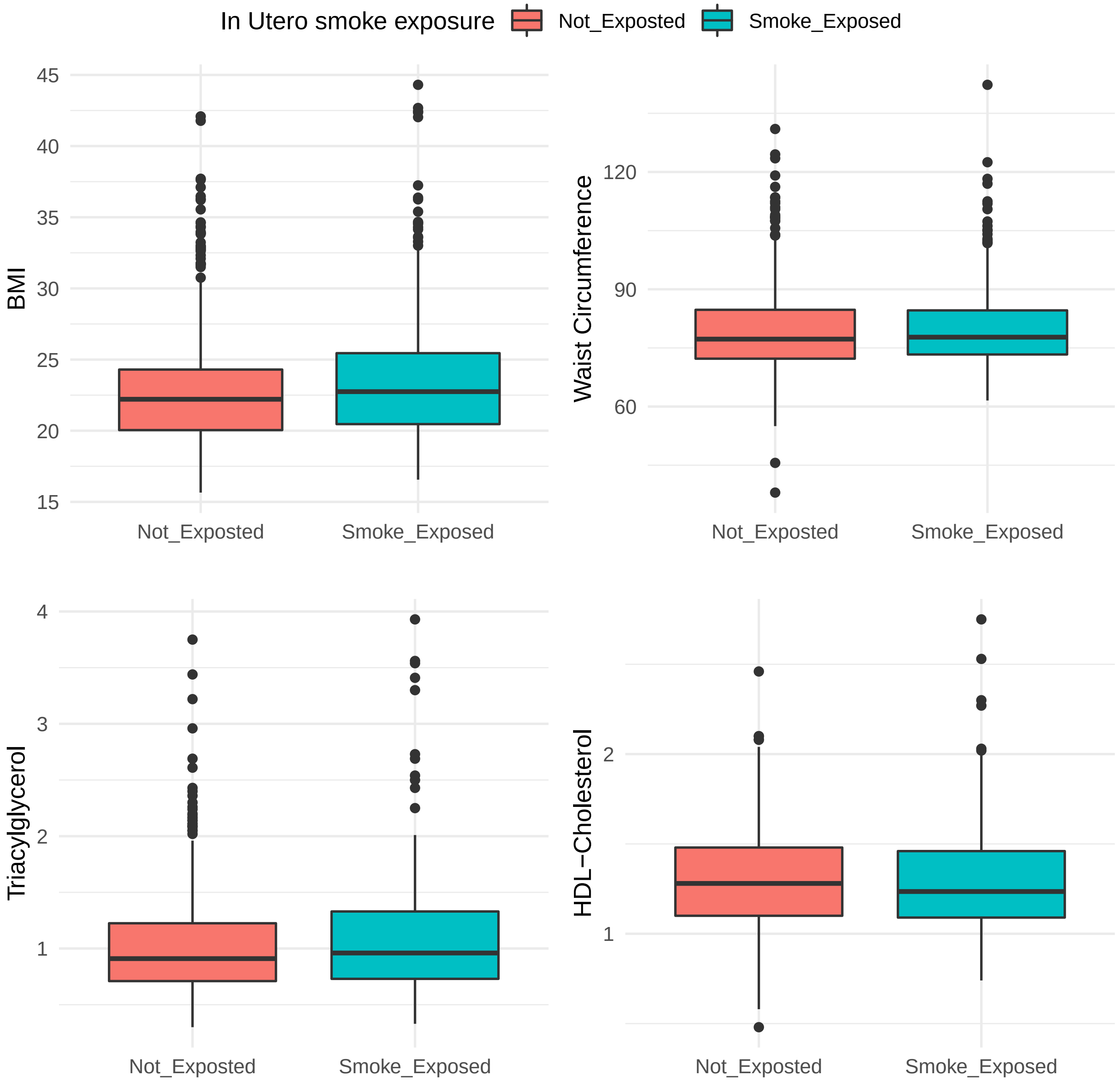
In Utero smoke exposure
Not_Exposted
Smoke_Exposed
BMI
45
40
35
30
25
20
15
Not_Exposted
Smoke_Exposed
Waist Circumference
120
90
60
Not_Exposted
Smoke_Exposed
Triacylglycerol
4
3
2
1
Not_Exposted
Smoke_Exposed
HDL−Cholesterol
2
1
Not_Exposted
Smoke_Exposed
